# Supplementary material for: Exploring areas of consensus and conflict around values underpinning public involvement in health and social care research: a modified Delphi study
Source: BMJ Open. 2014 Jan 10;4(1):e004217. doi: 10.1136/bmjopen-2013-004217 (PMC3902382; doi:10.1136/bmjopen-2013-004217)
Supplement: Author's manuscript [file bmjopen-2013-004217.draft_revisions.pdf]

Exploring areas of consensus and conflict around values underpinning public involvement in health and social care research: A modified Delphi study

Journal:	*BMJ Open*
Manuscript ID:	bmjopen-2013-004217
Article Type:	Research
Date Submitted by the Author:	10-Oct-2013

Complete List of Authors:	Snape, Dee; University of Liverpool, Public Health & Policy<br>Kirkham, Jamie; University of Liverpool, Biostatistics<br>Preston, Jennie; University of Liverpool, MRCN Community Liaison<br>Popay, Jennie; University of Lancaster, Division of Health Research<br>Britten, Nicky; University of Exeter, Institute of Health Service Research<br>Collins, Michelle; University of Lancaster, Division of Health Research<br>Froggatt, Katherine ; University of Lancaster, Division of Health Research<br>Gibson, Andy; University of Exeter, Institute of Health Service Research<br>Lobban, Fiona; University of Lancaster, Division of Health Research<br>Wyatt, Katrina; Peninsula College of Medicine and Dentistry,<br>Jacoby, Ann; University of Liverpool, Public Health & Policy

**Primary Subject Heading**:	Health services research
Secondary Subject Heading:	Research methods, Patient-centred medicine, Health policy, Evidence based practice, Ethics
Keywords:	Public involvement, Values, Conflict, Consensus, Health research, Delphi Technique

For peer review only - http://bmjopen.bmj.com/site/about/guidelines.xhtml

Exploring areas of consensus and conflict around values underpinning public involvement in health and social care research: A modified Delphi study

Snape D [a] , Kirkham J [b] , Preston J [c], Popay J [d], Britten N [e], Collins, M [d], Froggatt K [d], Gibson A [e], Lobban F [d], Wyatt K [e], Jacoby A [a] *

a Department of Public Health and Policy, University of Liverpool, Liverpool, UK
b Department of Biostatistics, University of Liverpool, Liverpool, UK
c NIHR MRCN Consumer Liaison Manager, University of Liverpool, Liverpool, UK
d Division of Health Research, Lancaster University, Lancaster, UK
e Institute for Health Research, Exeter Medical School, Exeter, UK
* Corresponding author E-mail address ajacoby@liv.ac.uk

Address for correspondence:
Ann Jacoby
Department of Public Health and Policy
University of Liverpool
Whelan Building
The Quadrangle
Brownlow Hill
Liverpool
L69 3GB
UK

Email: ajacoby@liv.ac.uk
Tel: 00 44 151 794 5602
Fax: 00 44 151 794 5588

***Key words*:**
Public involvement
Values
Conflict
Consensus
Health research
Delphi technique

For peer review only

ABSTRACT

Objective: There is a growing interest in the potential benefits of public involvement (PI) in health research. However, there has been little examination of the values underpinning PI or of how these values might differ for different stakeholder groups. Our objective was to explore areas of consensus and conflict around normative, substantive and process-related values underpinning PI in research.

Design: Mixed method, three-phase, modified Delphi study, conducted as part of a larger multi-phase project.

Setting: The UK health and social care research community.

Participants: Clinical and non-clinical academics, members of the public, research managers, commissioners and funders, identified via research networks, on-line searches and a literature review.

Results: We identified high levels of consensus across stakeholder groups for many normative, substantive and process-related issues explored. However there were also areas of conflict in relation to issues of bias and representativeness; and the normative issues around whether the purpose of PI in health and social care research is to bring about service change or generate new knowledge. There were large differences by subgroup in the percentages endorsing the ethical justification for PI; and the argument that PI equalises power imbalances. At an operational level, research support infrastructures were reported as lacking. Participants reported shortcomings in the uptake and practice of PI. Embedding PI practice and evaluation in research study designs was seen as fundamental to strengthening the evidence base.

Conclusions: Our findings about value consensus highlight the extent to which PI is already embedded in health and social care research. However they also highlight a need for 'best practice' standards to assist research teams to understand, implement and evaluate PI. These findings have been used in the development of Public Involvement Impact Assessment Framework (PiiAF), which offers guidance to both researchers and members of the public involved in the PI process.

ARTICLE SUMMARY

Strengths and limitations of this study

- Despite growing interest In the UK and internationally in the potential benefits of public involvement (PI) in research, there has been little examination of the values underpinning it, or of how these values might be different for different stakeholders. As part of a large study developing guidance on how to assess the impact of public involvement in research we undertook a mixed method modified Delphi study which has provided primary evidence of areas of consensus and conflict around the value systems underpinning (PI) in research.

For peer review only

- Our mixed method, modified Delphi study involved a heterogeneous panel of PI experts, reflective of the range of key stakeholder groups and was geographically diverse.
- ‘Consensus’ thresholds were determined in advance of data collection; with two levels of consensus defined.
- A limitation of our study was that response rates were low and that our conclusions are potentially biased, since panellist s may have been individuals with a strong commitment to PI. However, a large percentage of Round 1 survey responders also completed the Round 2 survey; and the quality of the data was high.
- Our findings provide important evidence about value consensus, but also highlight a need for ‘best practice’ standards to assist research teams in the PI process.

For peer review only

LAY SUMMARY

The purpose of this modified Delphi study was to examine areas of agreement and disagreement about the values underlying public involvement (PI) and the possible impacts of PI in health and social care research.

The study involved initial workshops with PI experts, to identify topics for exploration. Experts were representative of the various stakeholder groups subsequently recruited to a large-scale on-line survey. These were: members of the public involved in research; clinical and non-clinical researchers; research managers and research funders. Following the workshops, a pilot survey was conducted to develop and refine the survey questionnaires. Two survey rounds were then conducted, involving 318 respondents in round 1 and 231 in round 2. Both quantitative and qualitative responses were collected.

Overall, we identified high levels of agreement across the stakeholder groups for many of the issues explored. These areas of agreement reflect the extent to which PI is already embedded in health and social care research. However, we also found some areas of conflict between stakeholder groups.

Key findings in relation to the values underlying PI were -

- High levels of agreement that members of the public can and should influence how research is used
- Different and sometimes conflicting views across stakeholder groups about the possible biases and lack of representativeness in PI
- Different and sometimes conflicting views across stakeholder groups as to whether the purpose of PI in research is to bring about service change or generate new knowledge
- Agreement that there are tensions between what members of the public and researchers see as the purpose of research, and also what constitutes good research
- Stakeholder differences in the percentages endorsing the ethical justification for PI

Our findings highlight a need for 'best practice' standards to assist researchers when involving members of the public in their research projects. These findings have been used in the development of Public Involvement Impact Assessment Framework (PiiAF), which offers guidance to both researchers and members of the public involved in the PI process.

For peer review only - http://bmjopen.bmj.com/site/about/guidelines.xhtml

1. INTRODUCTION

There is a growing interest internationally in the potential benefits of Public Involvement (PI) in health research; for example, the Patient-Centred Outcomes Research Institute (2013) at the University of Pittsburgh and the Consumer and Participation Program (2013) taking place at the University of Western Australia. Within the United Kingdom, the involvement of members of the public in research is firmly established in health and social care policy (DoH, 2005; Myrhaug, 2006; Nilsen et al, 2009) and organisations including, for example, the National Institute for Health Research (NIHR, 2012) increasingly identify public involvement (PI) as a pre-requisite for funding.

There is a plethora of literature illuminating the experiences of members of the public and professional researchers, and the process of PI and its potential impacts (Beresford, 2002; 2003; Popay et al, 2007; Wyatt et al, 2008). However, while studies report on use of PI in context of priority-setting (Caron-Flinterman et al, 2005; Wright et al, 2007), the conduct of clinical trials (Marsden & Bradburn, 2004) and the identification of treatment outcomes (Hewlett et al, 2006), there has been relatively little examination of the values underpinning PI, or about capturing and assessing the impact of PI effectively (Minogue et al, 2005; Boote et al, 2002; Staley, 2009; Barber et al, 2011; Staniszewska, 2011). Possible reasons cited for the limited number of studies evaluating the impact of PI are: that evaluation is too difficult; and that PI is of intrinsic value and as such needs no further justification (Entwistle et al, 1998; Oliver et al, 2001; 2008, Staley K, 2009). The authors of the present paper acknowledge this latter perspective. However, an important challenge arises from the lack of an evidence base on the impact of PI, which we would argue is important to ensure integrity, avoid adverse effects and ensure PI is adequately resourced. The modified Delphi study reported here was part of a larger MRC funded study (G0902155/93948) which aimed to review evidence on the values and impacts associated with PI, develop guidance on how these impacts can be assessed and contribute to the development of good practice standards for PI. The modified Delphi study utilised mixed methods to explore areas of consensus and conflict underpinning three over-arching value systems identified from a preceding literature review (Gradinger et al, accepted for publication). These values relate to (i) normative perspectives on PI, which consider involvement as an end in itself, for example, rights and empowerment; (ii) substantive perspectives which consider the consequences of PI, for example quality and relevance, and (iii) process-related values associated with good involvement, for example, partnership and equality.

2. AIM AND OBJECTIVES OF THE DELPHI STUDY

The overall aim of the modified Delphi Study was to explore areas of consensus and conflict around the normative, substantive and process-related values underpinning PI in health and social care research. The specific objectives were:

For peer review only - http://bmjopen.bmj.com/site/about/guidelines.xhtml

- To explore key issues identified from our literature review with PI experts
- To provide new knowledge about the views of different stakeholder on PI
- To contribute to the development of guidance on how to assess the impact of public involvement in research.

3. METHODS

In this study, we defined PI as an active partnership between members of the public and researchers in the research process, rather than the use of people as the 'subjects' of research. The term 'public' has been used as developed by the UK National Advisory Group INVOLVE (2012a), and includes patients and potential patients, carers and people who use health and social care services. Within the study reported here, PI has been achieved through the collaboration of academic researchers with experience of working in the field of PI, user investigators and the wider project's Public Advisory Group whose members contributed to all five phases of the work. Approval for the study was given by the University of Liverpool Research Ethics Committee. A mixed methods approach to data collection was employed in a three-stage, modified Delphi process:

i) Three Expert Workshops (participant total n=42) including members of the public, academic and user-researchers, researcher/clinicians, research funders and research managers were conducted to generate qualitative data. The range of normative, substantive and process-related values underpinning PI, identified in a previously conducted literature review, (Gradinger et al, submitted for publication) were discussed, to identify concepts to be explored at Round 1 (R1) and Round 2 (R2) of the subsequent modified Delphi survey;

ii) A pilot study to test the R1 survey questionnaire. As a strategy to reduce attrition (Duffield, 1988; Hatton and Nunnelee, 1995), careful attention was paid to the content and layout of the invitation e-mail, the survey layout and the clarity of questions. Piloting was conducted with academic (n=6) and user-researchers (n=3) and members of our Patient Advisory Group (n=2). Language, question type and questionnaire formatting were edited in response to participant feedback;

iii) A two-round modified Delphi survey was undertaken between November 2011 and September 2012 to explore areas of consensus and conflict around the normative, substantive and process-related values underpinning PI in health and social care research. In this paper we describe the modified Delphi process and report on the findings relating to the values underpinning PI in health and social care research. A subsequent paper will report the survey findings in relation to perceived PI barriers and facilitators, perceived impacts of PI and potential approaches to its evaluation.

A modified Delphi technique

The main premise of the Delphi technique is the assumption that group opinion is more valid than individual opinion. The technique offers a reliable data collection method in circumstances where there is uncertainty or a paucity of knowledge surrounding the topic

For peer review only

area under investigation (Dalkey & Helmer, 1963; Gallagher et al, 1993; McKenna, 1994; Crisp et al, 1997; Hassen, 2000). While the Delphi technique has been used extensively within health and social science research (Jones & Hunter, 1995; Walker & Selfe, 1996; Keeney et al, 2001; Campbell et al, 2002; Daykin et al, 2002; Sheild et al, 2003; Boote, 2006; Efstathiou et al, 2008), criticism of the technique includes the perception that it seeks to force consensus and so is weakened by not allowing panellists to elaborate on their views (Walker & Selfe, 1996). In response to this criticism, the current study used a modified Delphi technique which did not force consensus; rather panellists were provided with opportunities to elaborate on why they held the views they expressed or endorsed (Keeney et al, 2001) in order to allow us to try and tease out areas of conflict as well as areas of consensus.

There are a number of key concepts and assumptions which distinguish the basic Delphi technique from other research methods. These include anonymity, multi-stage iteration and controlled feedback, exploration of consensus via statistical group response and the use of experts (Lynn et al., 1998; Hasson et al, 2000). Each of these key characteristics was given due consideration as a means of enhancing the validity and reliability of the study design and the quality of responses (Linstone and Turoff, 1975; Sackman, 1975; McKenna, 1994; McDonnell et al, 1996; Hardy et al, 2004).

Sampling and the use of 'experts'

To reduce possible bias, our sampling strategy for panel composition was both reflective of the population subgroups under investigation and geographically diverse (Jones & Hunter, 1995; Keeney et al, 2001). To increase the reliability of the results a large heterogeneous panel of 'experts', consisting of distinct stakeholder groups was recruited (Duffield, 1993). We defined 'experts' as a group of *informed individuals* (McKenna, 1994) or those with knowledge or experience of a specific subject (Lemmer, 1998; Green et al, 1999). Sampling was purposive. We aimed to capture the various PI perspectives and interests of members of the public, user, academic and clinical researchers, and research managers, directors, commissioners and funders. Potential panellists were identified in one of three ways: directly, through research team members' contacts and networks; through on-line searches of open-access research information and funding sites; and via a review of literature in the field of PI in health and social care research.

Anonymity

Complete anonymity could not be achieved as survey panellists and their responses were known to some members of the research team. However, anonymity between panellists was guaranteed. Reactions to panellists' opinions, key arguments and levels of consensus for each sub-group were fed back to panellists at R2 of the modified Delphi survey; each opinion carried the same weight and was afforded the same degree of importance in the analysis. In this way, subject bias was eliminated (Keeney et al, 2001). This approach also enabled panellists to be open and honest about their views on various issues as well as providing

For peer review only

them with an equal opportunity to express an opinion without feeling pressured psychologically to conform to the views of others (Keeney et al, 2001).

Quantitative data analysis

Determining how consensus cut-off points are reached and understanding how they are derived is often problematic, as the reporting of such criteria is limited within many published studies (Crisp et al, 1997). To address this, we sought statistical advice and specific criteria for determining consensus thresholds were applied in advance.

We took, as representing **clear** consensus (in either a positive or negative direction) around a statement, endorsement of the corresponding response categories/scores (strongly agree/agree; strongly disagree/disagree) by at least 60% of respondents. We took, as representing **critical** consensus (in either a positive or negative direction) around a statement, endorsement of the corresponding response categories/scores by at least 70% of respondents, with at least 55% endorsing the extreme categories in the scales (i.e. strongly agree/disagree; major barrier/facilitator). Where an item reached the level specified above as 'clear consensus', attention was paid to the distribution of responses of the remaining 40%. Where these were evenly distributed across the remaining response options, the definition of consensus was accepted. Where responses cluster in one response option only, consensus was not assumed and this item was further explored in R2 of the survey.

At R2 the results from the R1 survey were fed back to panellists in the form of bar charts, textual summaries of statistical data and summarised descriptions of qualitative findings. Feedback at R2 included comparison of panellists' own subgroup responses with those of the other sub-groups for each item. Particular attention was drawn to items demonstrating within-subgroup dissent, and within-subgroup consensus but between-subgroup dissents. Items at R1 where responses for all participants as a group reached the level defined for clear consensus (i.e. 60%), but the between-subgroup percentage differences for consensus were large (i.e. more than 10% difference in percentages endorsing agreement/disagreement between subgroups), were explored in R2. Also explored at R2 were any 'unexpected' (in the collective view of the study team) endorsements of items by the subgroups. We draw attention to these 'unexpected' endorsements in the findings section below.

Qualitative data analysis

In both R1 and R2, to enable in-depth exploration of the quantitative findings, panellists were provided with the opportunity to make further comments as they saw fit, through open questions. Thematic codes were identified using Framework Analysis (Ritchie & Spencer, 1994), a matrix based method for ordering and synthesising data. The analysis was conducted by DS. Quality checking of the coding process and reduction of coding bias was ensured by AJ who reviewed 10% of the qualitative data. Firstly, data were reviewed inductively to identify recurring themes and concepts raised by panellists. These were coded

For peer review only - http://bmjopen.bmj.com/site/about/guidelines.xhtml

and formed the initial major and sub themes; additional codes were then incorporated through an iterative process involving DS and AJ. The thematic framework was further refined before being applied systematically to the whole data set. This process facilitated the identification of any inconsistencies in coding which were subsequently discussed and reconciled.

4. RESULTS

The iterative nature of the Delphi technique enabled R2 of the survey to be informed by the results of R1. For this reason, the methods and results for both rounds are discussed together.

Panellists

At R1 seven hundred and forty (n=740) potential 'expert' panellists were invited, via e-mail, to participate in the on-line survey. Non-responders were e-mailed up to two reminder letters as appropriate, yielding a total response of three hundred and eighteen (n=318) (response rate 43%). Responding panellists self-selected themselves into one of five 'stakeholder' groups: clinical academic [CA], (20%); non-clinical academic [NCA], (28%); member of the public [MP], (17%); research manager or funding / commissioning body employee [RM], (24%); or, occupying multiple roles [MR], (11%).

Analysis of the characteristics of those who took part in R1 confirmed the high level of expertise among panellists. Most had at least five years experience in research, with three-quarters having some responsibility for PI. At R1 panellists reported being involved in a wide range of research roles including, for example: drafting research proposals and protocols (n=239; 86%); developing plain English summaries (n=238; 86%); sitting on project advisory and steering groups (n=228; 82%); presenting research findings (n=226; 82%); and being involved in data collection (n=223; 81%).

However, despite this high level of expertise, fewer than half (n=134; 48%) of the panellists had training relevant to PI in health and social care research; rather they 'learned by doing'. Panellists cited a number of practical learning opportunities which underpinned their PI skills acquisition; including, for example:

> *"Good networking".* [NCA, R1]
>
> *"…hard work building rapport and understanding of the patients' perspective, putting myself in their shoes".* [NCA, R1]
>
> *"Support from others, learning alongside others, and time".* [CA, R1]
>
> *"…so learning from mistakes and trying to listen to what both the patients and the researchers need to make it a meaningful experience".* [MP, R1]
>
> *"Various policy documents for engaging the public in research on which we can rest our local framework and priorities".* [RF, R1]

All panellists (n=318; response rate 43%) who submitted the R1 questionnaire were eligible to participate in R2. Of the 318 responders, three electronically 'opted out' of

For peer review only - http://bmjopen.bmj.com/site/about/guidelines.xhtml

receiving further e-mail communications. The R2 questionnaire was therefore e-mailed to three hundred and fifteen (n=315) panellists. Up to two reminders were actioned as required. A total of two hundred and thirty one (n=231) R2 survey responses were received across the 5 stakeholder groups: clinical academic [CA], (17%); non-clinical academic [NCA], (29%); member of the public [MP], (18%); research manager or funding / commissioning body employee [RM], (24%); and, occupying multiple roles [MR], (12%); yielding a R2 response rate of 73% (of the 43% who took part in R1).

Arguments related to the nature of knowledge

We asked our panellists to comment on what kinds of knowledge they believed should inform health and social care research. Responses at R1 (see Figure 1) revealed critical consensus across the different stakeholder groups (over 90% strongly agreed/agreed) for the substantive argument that members of the public have unique knowledge and expertise that is complementary to that of professionals/clinicians and researchers, and should be valued equally.

A comment from one clinical academic panellist illustrates this:

> *"It is important not to conflate the complementary perspectives of clinical / health, social care professional researchers versus methodologists. Both have important knowledge and expertise to bring to the table in informing health and social care research, as do patients, their informal carers and advocates and members of the public…it is important that all of these perspectives are regarded as equally important, rather than privileging any one view over the others".* [CA; R1]

Though it just failed to reach our definition of clear consensus, there was broad support across the stakeholder groups (58%) for the idea that while professionals/clinicians and researchers may also be service users, they cannot themselves represent user issues effectively. Since there was also some support across stakeholder groups (just over 50% strongly agreed/agreed) for the idea that both members of the research community and members of the public are likely to have biased views about research, we explored this issue of bias further in R2.

[Insert Figure 1]

At R2 we asked panellists to comment on whether they felt it mattered if different stakeholder groups held views which others considered biased. Forty three percent (43%) said "*yes"* they did feel it mattered, with a percentage range of 29%-59% across stakeholder groups. Individual stakeholder group responses are outlined in Table 1. [Insert Table 1]

A common theme across panellists' written comments and one articulated by a panellist holding multiple roles, was that all research actors are biased to some degree; holding different views based on knowledge and / or experience:

For peer review only - http://bmjopen.bmj.com/site/about/guidelines.xhtml

> *"All views will have a bias of some kind, but I think it is more important that the view is 'informed'. Bringing together the different perspectives in an open, collaborative way is one of the strengths of patient involvement, not a weakness".* [MR, R2].

Many panellists articulated that differences in perspective should not necessarily be viewed negatively; rather they should be acknowledged as a "*given*". Interestingly, at R2 the issue of power (who holds the power to enforce their viewpoint) was highlighted as more important than the question of bias.

Following from this, in R2 process values that involve *"building relationships of trust between the different stakeholder groups"* [NCA, R2], were frequently argued to be key for managing difference and essential for effective stakeholder partnerships and shared decision making.

Arguments related to the purposes of PI in health and social care research

There was critical consensus (over 80% strongly agreed/agreed) at R1, across all stakeholder groups, that members of the public should be involved in publicly funded research, and are entitled to say what and how research is undertaken (see Figure 2). There was also critical consensus that members of the public should be involved in research impacting on their own health or on NHS functioning; and clear consensus that they can influence how such research is used (see Figure 2). However, over 60% agreed/ strongly agreed that there are tensions between what members of the public and researchers see both as the purpose of research and what constitutes good research.

[insert Figure 2]

At R2 we asked panellists to comment on whether they believed these/such tensions within the arena of health and social research could be resolved. Given that tensions between the different stakeholder groups in the PI enterprise have been highlighted elsewhere (Boote et al, 2010), a somewhat unexpected finding from our data was critical consensus (75%) that tension resolution was possible. The percentage range across stakeholder groups was 67%-89%. Individual stakeholder group responses are outlined in Table 2.

[Insert Table 2]

Also unexpected was that, though tension was seen as an inevitable consequence of potential differences in perspective and/or expectations, it was not necessarily perceived as a negative issue. Rather it was seen by many as a means to stimulate critical debate:

> *"There should be tensions because that indicates passion; but the differences need to be managed in an open and transparent manner".* [MP, R2]

> *"Tensions are a natural consequence of different stakeholders having different priorities. To some extent the debate they lead tp could be regarded as conducive to better decisions.' [NCA, R2]*

> *"This is something to value, rather than to be concerned about! As long as those considering the various perspectives understand in advance that there may be differences in opinion, then there aren't 'tensions', just differences." [CA, R2]*

> *"…if researchers, clinicians and members of the public work together at the start of a trial, it will significantly reduce these tensions. However I also think that you can never entirely remove the tensions, and to some extent this is a good thing as the element of struggle helps to refine the research question."* [MP, R2]

Once again, the need to promote values of effective partnership working - including, for example, reciprocity, communication, reflexivity and learning from each other - was seen as key to tension resolution over time

There was support in panellists' written responses at R1 for the idea that PI training for members of the public needed to not only include an overview of research design and methods but should also involve education about political and policy context(s) which would lead to greater understanding of fundamental research drivers and processes. It was articulated by some panellists that training could also increase empowerment of members of the public, promoting and facilitating partnership working within the research team. However, panel members also reflected that this should not stop academic researchers from recognising and valuing the experiential knowledge of members of the public themselves.

Arguments relating to the why and the how of PI

There was overall critical consensus (over 70% strongly agreed/agreed) that research is more ethical when members of the public are involved at all stages of the process. However, this statement was most strongly endorsed by members of the public (94% strongly agreed/agreed) and least endorsed by clinical (67%) and non-clinical (59%) academics. There was critical consensus across stakeholder groups (81% strongly agreed/agreed) that involvement is empowering for members of the public. Again, members of the public and those with multiple roles were much more likely to agree (75% strongly agreed/agreed) than were other groups (54%) that PI equalises the power between them and professionals. Clear consensus was also reached (over 65% agreed strongly/agreed) in relation to the statement that PI was often a "*tick the box*" exercise, with members of the public more likely to express disappointment at the lack of opportunity to influence the research process. Some panellists (across all stakeholder groups) suggested that involvement was still often seen as being tokenistic; with "*lip service*" only paid to the issue of PI:

> "*There is the perception that it is an inconvenient tick box exercise giving no added value and actually slowing research process down"* [CA, R2].
>
> *"Until researchers recognise the importance and value of public involvement, and how it can improve the quality of their research, they are likely to give it low priority"* [CA, R2].
>
> *"While PI is no longer an optional it can still feel like a hassle for the academic community with gains that can seem remote. That needs to change otherwise people will continue being tokenistic*" [NCA, R2]

Panellists voiced a number of concerns about involving members of the public in the research process, including the lack of funding to involve the public in the early stages of

For peer review only - http://bmjopen.bmj.com/site/about/guidelines.xhtml

research question and proposal development. Time pressures related to grant applications and project-specific time-lines were also highlighted as problematic, including lack of time to take account of public members' illness and/or work commitments, the lack of realistic timeframes for reviews and, not least, the lack of time needed to develop and nurture research team relationships.

At R1 a number of panellists expressed the view that a "*fit-for-purpose*" model of PI, which reflected study and social contexts, was a more appropriate way of assuring effective PI than the various "*levels of PI"* frameworks proposed in the PI literature (see for example, Hanley et al 2004). Such frameworks were not popular with panellists, who argued that the hierarchical approach they embody to articulate different PI levels was misleading and not necessarily an indication of PI quality. The framework approach to PI was further criticised for not addressing all the facets of involvement, and as such having limited application, and becoming potentially a driver for tokenism. Interestingly, INVOLVE (2012b) have recently updated their guidance to refer to 'approaches' rather than to 'levels' of involvement and also recognises that different approaches to PI might be used within the same project.

Following these responses we asked panellists at R2 whether, in their opinion, they felt there were any circumstances where PI was inappropriate. There was critical consensus among panellists that members of the public could be involved in all/any type of research - with the caveat that PI may be more challenging within the field of basic science research. The following observation made by one panellist, a research manager/ funder, serves to illustrate the potential for variation in the way members of the public can be involved:

> *"…hard to imagine a rationale for no involvement, but it will vary from project to project. One argument is often made that there shouldn't be public involvement in pure basic biomedical research and certainly the level of involvement will be very different to that, say, of a clinical trial. But there is still potential for involvement for example in wider public debate/involvement about evaluating this type of research and making policy decisions about how much money to invest in this type of research than more applied research. Some scientific projects may require human samples and there is a role for the public in the governance issues around this type of research - or motivating others to donate tissue etc. I have come across some aspects of clinical trials where, for example, two different scales for measuring depression were compared - a very technical and limited bit of work - with no room for influence from public involvement - but this was one part of a much larger trial where there was plenty of scope for involvement. So it may not always be appropriate for public involvement to influence the design and delivery of a project if it is very technical - but there is likely to be value of involvement in other aspects of the project around governance and funding decisions and perhaps dissemination in informing people that the research is going on - and explaining the implications of the research to a lay audience"* [RM, Round 2].

5. DISCUSSION

This paper reports findings from an on-line, modified Delphi study involving a range of stakeholder groups to explore normative, substantive and process-related values underpinning PI in health and social care research. This approach to gathering experts' opinions and suggestions without the need to bring them together physically, proved both

For peer review only - http://bmjopen.bmj.com/site/about/guidelines.xhtml

versatile and resource-efficient. In this study, Delphi panellists' responses were fairly evenly balanced across the various stakeholder groups; and the response rate was, in our view, acceptable given the 'cold call' approach (Sitza & Wood, 1998; Arber, 2001; Edwards et al, 2008). Allowing panellists an opportunity to comment on their interpretation of the items and to express their views via open-question feedback increased the reliability of the study and improved the validity of the results (McDonnell et al, 1996).

In the following discussion, we first consider key themes emerging from the data in relation to the normative, substantive and process-related values underpinning PI in health and social care research; second, we highlight the potential limitations of our research approach; and last, we present our conclusions.

Key themes

Our findings suggest high levels of self-reported expertise amongst members of the public involved in health and social care research, regardless of little 'formal' education and training in PI being reported. Panellists reported that timely and appropriate training is required by members of the public to support their involvement. Similarly, panellists also articulated that other actors in the research process require training in how to engage and support members of the public in their research roles. Support strategies suggested by panellists included, for example, advice and mentoring schemes.

Overall, our R1 survey identified high consensus across stakeholder groups for many of the normative, substantive and process-related issues explored. These included: that members of the public have an entitlement to be involved in the research process, including in consideration of what research is undertaken and how it is used: and that different types of knowledge are important and that members of the public have unique knowledge. These areas of consensus highlight the extent to which PI is embedded in health and social care research. However, there were also areas of conflict by stakeholder group, for example there was wide variation in the percentages endorsing the normative position that research is more ethical where there is PI throughout. Although ethical concerns are paramount in the context of health and social care research, it is interesting to note that the two stakeholder groups least likely to endorse the importance of PI to ensure research probity were clinical and non-clinical academics. There was also disagreement about the extent to which PI was seen as equalising power between public and professionals; and about the substantive issues around potential stakeholder group bias and representativeness.

Most health and social care research areas, including pre-clinical, were deemed appropriate for PI; with many panellists articulating their views about how and when it might be feasible to introduce and evaluate involvement. This reflects recent changes in commitment by UK funding bodies to incorporating PI across all research designs and at all stages of the research process (NIHR, 2012). Assessments about the appropriateness of PI need to be viewed within the overall opportunities for members of the public to influence project-specific agendas. A recent review of case studies conducted by Boote and

For peer review only - http://bmjopen.bmj.com/site/about/guidelines.xhtml

colleagues (2010) highlighted potential tensions between different stakeholder groups involved in PI. . However in our study the potential for tension was articulated as an inevitable consequence of collaborative working; individuals' agendas can be different when designing research studies. The acknowledgement and management of this standpoint was deemed essential in reconciling potential stakeholder difference. By promoting values of effective partnership including, for example, communication, reflexivity and learning from each other, research relationships can develop overtime, and tension can lessen as individuals worked towards a common goal. The recognition that tensions are inevitable and can be used productively through questioning and discussion has also been highlighted by Abma and Widdershoven (2005).

It is interesting to note that although panellists recognised the normative debates around involving members of the public in health and social care research and acknowledged significant progress in doing so, they also reported shortcomings in process and substantive-related issues around the practice of PI in research, not least of which is the need for quality standards wherein involvement is embedded methodologically in research study designs. Promoting and evaluating PI as 'normalised' research practice was seen as key to strengthening the evidence base around PI which in turn, it was argued, would reduce the practice of tokenism.

Delphi study limitations

In this study, we chose to adopt a modified Delphi approach for data collection. This involved the use of both fixed choice and open questions in order to try to maximise our understanding of the issues under consideration. As with all survey approaches, there are inevitably limitations to the depth of the data obtained; and it could be important to follow up key issues using more in-depth approaches, which would allow for more detailed exploration of less well understood and articulated issues.

Although Delphi techniques vary, face-to-face contact with participants at round one has been found useful in increasing the response rate (McKenna, 1994). However, due to the size of our sample, many of the panellists were targeted 'cold' without any prior notices. This approach may have had an impact on our response rate at R1. The use of reminders is generally endorsed in texts on survey methods (McColl et al, 2001) and in line with Peterson and colleagues (1989), two reminder cover letters were e-mailed to non-responding participants at both R1 and R2 of the survey to stimulate additional responses.

That our response rate to Round 1 was low was unsurprising given our approach; however, it was encouraging that a large percentage of responders to Round 1 subsequently completed Round 2.The Delphi technique requires continued commitment from participants throughout the data collection process. Consideration must be given to the fact that individual time constraints and lack of familiarity with the Delphi technique may have prevented some participants from being able to make such a commitment. Nonetheless, the quality of the

For peer review only

responses provided made clear that those who did take part were firmly committed to offering us detailed and extremely thoughtful answers to our questions.

Another potential limitation relates to the representativeness of our panellists. Under half of those we approached participated in Round 1 of the study, and this proportion was further reduced at Round 2. Since those who opted in to the survey self-selected the stakeholder group with which they aligned themselves, we have no information about the groupings of those who opted out. We also have no information about other characteristics of interest for which we collected information from participants, for example, years of involvement in PI or extent of training in PI. We are therefore unable to comment meaningfully on the representativeness or otherwise of the study population. A further limitation arising from this is that those opting to take part in the Delphi study may have been individuals with a particularly strong commitment to PI in research, who were therefore keen to endorse its validity; and our findings may therefore offer an overly optimistic picture. This needs to be borne in mind when interpreting the findings.

Conclusions

This study has identified a number of key issues around PI implementation, in relation to potential value conflict. This study serves as one of the few attempts to empirically gauge the views of key stakeholders on these issues. The findings suggest that there is a large degree of consensus across many of these issues, but also a healthy level of debate in some important areas. While acknowledging the problem of representativeness of our panellists we feel that this Delphi survey provides us with important insight into the views of this group of PI experts.

Our findings also support the conclusion of Boote and colleagues (2012) that there is a need for PI best practice standards which research teams can follow when seeking to involve members of the public in the research process. The aim of the wider MRC research within which this Delphi study sits was to develop and pilot guidance document to support assessment of the impacts of PI and to produce draft best practice standards. Findings from our modified Delphi study about commonly held PI values and about assessment of PI impacts (the latter is the subject of a separate paper) have contributed to this wider work by identifying areas where conflict is likely to arise and suggesting ways such conflict can be negotiated so the PI agenda can move forward meaningfully.

For peer review only - http://bmjopen.bmj.com/site/about/guidelines.xhtml

Acknowledgements:

The authors of this paper are members of PiiAF research team, which involves researchers at the Universities of Lancaster, Exeter and Liverpool. Other members of the PiiAF team who contributed to the Delphi Study were: Felix Gradinger, Elaine Hewis, Tim Rawcliffe and Paula Williamson. Gradinger reviewed and commented on the survey questionnaires in light of the literature review he conducted as part of the wider MRC Study; Hewis and Rawcliffe took part in the expert workshops; Williamson contributed statistical expertise, including on the definition of consensus.

We would also like to acknowledge the valuable input of the members of the Public Advisory Group connected to this study: Bert Green, Faith Harris-Golesworthy, Dina Lew, Irene McGill, Nigel Pyart; and members of the Advisory Network. We are indebted to all those who took part in the Delphi study and wish to thank participants for their time and insight.

Funding statement:
The study was supported by the Medical Research Council's Methodology Research Programme [G0902155/93948]

Competing interests statement:

Professor Jennie Popay was a member of the MRC Methodology Research Programme at the time this grant was awarded, though had no involvement in the funding decision. There were no competing interests for the other authors.

Author contributions:

Snape was responsible for day-to-day management of the Delphi study, the qualitative data analysis and the drafting of the manuscript; Kirkham was responsible for management and analysis of the quantitative data; Preston assisted in the expert workshops, in development of the survey questionnaires and commented on the manuscript; Popay and Britten contributed to the conceptual development of the Delphi study and commented on the manuscript; Collins, Froggatt, Lobban and Wyatt all commented on the survey documents and the manuscript; Gibson assisted with the expert workshops, in development of the questionnaires and commented on the manuscript; Jacoby had responsibility for the overall conceptual and methodological development of the Delphi study, supervision of Snape, and drafting/finalising of the manuscript.

Data sharing statement:

We are exploring the scope for making the anonymised data files available from the corresponding author at the University of Liverpool, which would provide a permanent, citable and open access home for the dataset.

For peer review only

Figure 1: What kinds of knowledge should inform health and social care research?*

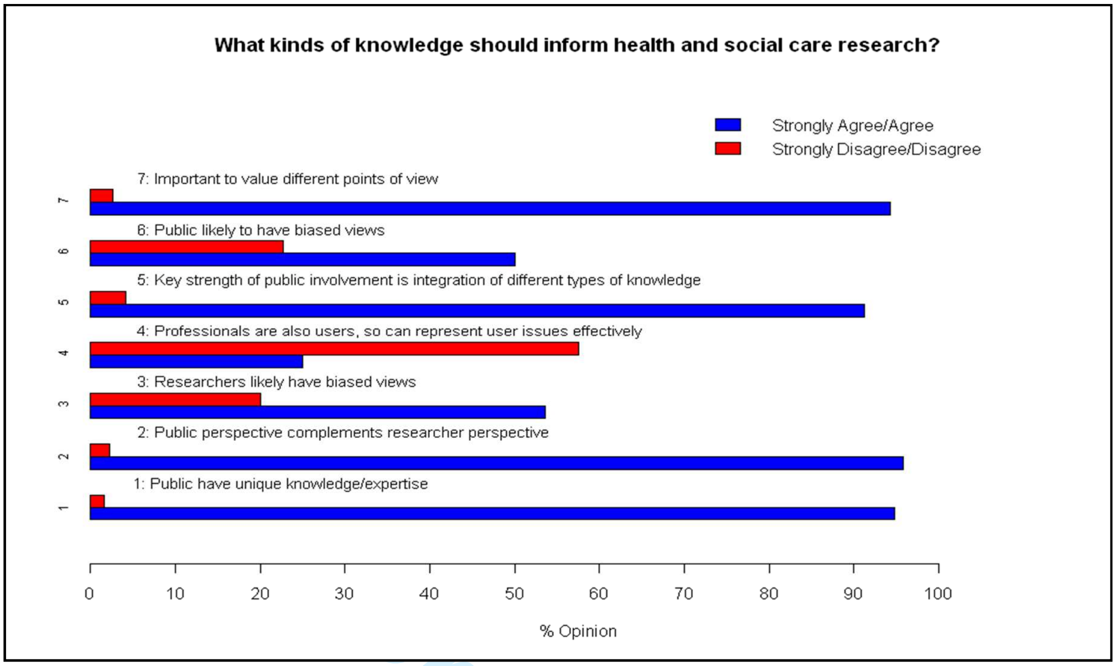

*Data taken from R1

For peer review only - http://bmjopen.bmj.com/site/about/guidelines.xhtml

Table 1: Does it matter if stakeholder groups hold views considered biased by others?*

Stakeholder Group	n	Bias Matters	Bias Doesn't Matter
Clinical Academic Researcher	39	23 (59%)	16 (41%)
Non-Clinical Academic Researcher	66	32 (48%)	34 (52%)
Member of the Public	41	17 (41%)	24 (59%)
Research Manager/Funder	56	16 (29%)	40 (71%)
Dual Role	27	10 (37%)	17 (63%)

* Data taken from R2

For peer review only

Figure 2: What are the purposes of public involvement in health and social care research?*

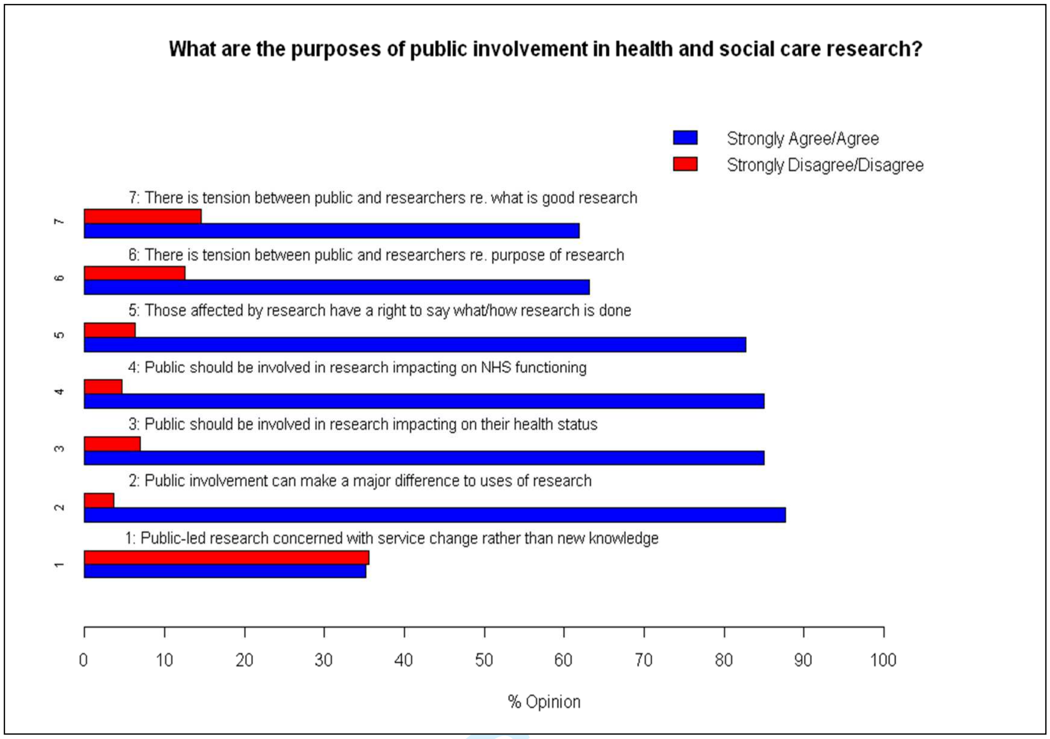

*Data taken from R1

Table 2: Can tensions be resolved in health and social care research?*

Stakeholder Group	n	Tension Resolution YES	Tension Resolution NO
Clinical Academic Researcher	38	34 (89%)	4 (11%)
Non-Clinical Academic Researcher	66	44 (67%)	22 (33%)
Member of the Public	41	35 (85%)	6 (15%)
Research Manager/Funder	54	37 (69%)	17 (31%)
Dual Role	26	18 (69%)	8 (31%)

*Data taken from R2

For peer review only

References

1. Abma T.A., Widdershoven G.A.M. (2005). Sharing stories. Narrative and dialogue in responsive nursing evaluation. *Evaluation and the Health Professions. 28; 90-109.*

2. Arber S. (2001). Designing samples. In: Gilbert N, ed. *Researching Social Life*. SAGE Publications: London.

3. Barber R., Boote J.D., Parry G.D., Cooper C.L., Yeeles P., Cook S. (2011). Can the impact of PI on research be evaluated? A mixed methods study. *Health Expectations.* Doi:10.1111/j.1369-7625.2010.00660x

4. Beresford P. (2003). User Involvement in research. *Nursing Times Research* 8:36; 36-46.

5. Beresford P. (2002). User Involvement in research and evaluation: Liberation or Regulation? *Social Policy & Society* 1:2; 95-105.

6. Boote J., Baird W., Sutton A. (2011). Public involvement in the systematic review process in health and social care: A narrative review of case examples. *Health Policy.* 102: 105-116.

7. Boote J., Baird W., Beecroft C. (2010). Public involvement in the design stage of primary health research: A narrative review of case examples. *Health Policy. 95;* 10-23.

8. Boote J., Barber R., Cooper C.. (2006). Principles and indicators of successful consumer involvement in NHS research: Results of a Delphi study and subgroup analysis. *Health Policy* 75:3; 280-297.

9. Boote J., Telford R., Cooper C,. (2002). Consumer involvement in health research: a review and research agenda. *Health Policy*. 61:2; 213-236

10. Campbell S.M., Braspenning J., Hutchinson A., Marshall M. *(2002).* Research methods used in developing and applying quality indicators in primary care. *Quality and Safety in Health Car.e* 11; 358–364.

11. Caron-Flinterman J.F., Broerse J.E.W., Teerling J., Bunders J.F.G. (2005). Patients' priorities concerning health research: The case of asthma and COPD research in the Netherlands. *Health Expectations* 8;3; 253-63.

12. Consumer and Community Participation Policy. University of Western Australia (2013). http://www.sph.uwa.edu.au/ ; 2013

13. Crisp J., Pelletier D., Duffield C., Adams A., Nagy S. (1997). The Delphi Method? *Nursing Research* 46:2; 116-118.

14. Dalkey N., Helmer O. (1963). Delphi Technique: characteristics and sequence model to the use of experts. *Management Science* 9:3; 458-467.
15. Daykin N., Sanidas M., Barley V., et al.(2002). Developing consensus and interprofessional working in cancer services: the case of user involvement. *Journal of Interprofessional Care* 16:4; 405–406.
16. Department of Health (2005). *Research Governance Framework for Health and Social Care.* 2nd Edition. Department of Health.
17. Duffield C. (1993). The Delphi Technique: a comparison of results obtained using two expert panels. *International Journal of Nursing Studies* 30:3; 227-237.
18. Edwards P, Roberts I, Clarke M et al. (2002). Increasing response rates to postal questionnaires: systematic review. *Br Med J; 324: 1183.*
19. Efstathiou N., Ameen J., Coll A.M. (2008). A Delphi study to identify healthcare users' priorities for cancer care in Greece. *European Journal of Oncology Nursing* 12:3; 262–371.

20. Entwistle V.A., Renfrew M.J., Yearley S., Forrester J., Lamont T. (1998). Lay perspectives: advantages for health researchers. *British Medical Journal* 316; 463-466.

21. Gallagher M., Hares T., Spencer J., Bradshaw C., Webb I. (1993). The nominal group technique: a research tool for general practice. *Family Practice* 10:1; 76-81.

22. Gradinger F, Britten N, Wyatt K, Froggatt K, Jacoby A, Lobban F, Mayes D, Snape D, Rawcliffe T, Popay J. Values associated with Public Involvement in Health and Social Care Research. Accepted for publication

23. Green B., Jones M., Hughes D., Williams A. (1999). Applying the Delphi technique in a study of GP's information requirement. *Health and Social Care in the Community* 7:3;198-205.

24. Hardy D.J., O'Brien A.P., Gaskin C.J., O'Brien A.J., Morrison-Ngatai E., Skews G., Ryan T., McNulty N. (2004) *Journal of Advanced Nursing* 46:1; 95-109.

25. Hassen F., Keeney S., McKenna H. (2000). Research Guidelines for the Delphi SurveyTechnique. *Journal of Advanced Nursing.* 32:4; 1008-1015.

26. Hewlett S., de Wit M., Richards P., Quest E., Hughes R., Heiberg T., et al. (2006). Patients and professionals as research partners: Challenges, practicalities, and benefits. *Arthritis & Rheumatism-Arthritis Care & Research* 55:4; 676-680.

27. Hanley B., Bradburn J., Barnes M., Evens C., Goodare M., Kelson M., et al. (2004). *Involving the public in NHS, public health and social care research: briefing notes for researchers*, 2nd Edition. Eastleigh. INVOLVE.

28. INVOLVE (2012a). *INVOLVE Strategy 2012-2015. Putting people first in research*. INVOLVE, Eastleigh [on-line] last accessed 18/06/2012 at URL http://www.invo.org.uk

29. INVOLVE (2012b) *Briefing notes for researchers: involving the public in NHS, public health and social care research*. INVOLVE, Eastleigh [on-line] last accessed 8/07/2013 at URL http://www.invo.org.uk

30. Jones J., Hunter D. *(1995).* Consensus methods for medical and health services research. *British Medical Journal* 311; 276–380.

31. Keeney S., Hassona F, McKennab H.P. (2001) A critical review of the Delphi technique as a research methodology for nursing. *International Journal of Nursing Studies* 38:2; 195-200.

32. Lemmer B. (1998). Successive surveys of an expert panel: research in decision making with health visitors. *Journal of Advanced Nursing* 27:3; 538-545.

33. Linstone H.A., Turoff M. (eds). (1975). *The Delphi Method: Techniques and Applications.* Addison-Wesley. Reading, Mass.

34. Lynn M.R., Layman E.L., Englebardt S.P., (1998). Nursing administration research priorities: a National Delphi Study. *Journal of Nursing Administration* 28:5; 7-11.

35. Marsden J, Bradburn J. (2004). Patient and clinician collaboration in the design of a national randomised breast cancer trial. *Health Expectations* 7; 6-17.

36. McColl E., Jacoby A., Thomas L., Soutter J., Bamford C., Steen N., Thomas R., Harvey E., Garratt A., Bond J. (2001). Design and use of questionnaires: a review of

For peer review only

best practice applicable to surveys of health service staff and patients. *Health Technology Assessment* 5:31;101-132.

37. McDonnell J., Meijler A., Kahan J.P., Bernstein S.J., Rigter H. (1996). Pannellist consistency in the assessment of medical appropriateness. *Health Policy* 37; 139-152.

38. McKenna H.P., (1994). The Delphi technique: a worthwhile approach for nursing? *Journal of Advanced Nursing* 19:6; 1221-1225.

39. Minogue V., Boness J., Brown A., Girdlestone J. (2005). The impact of service user involvement in research. *International Journal of Health Care Quality Assurance*. 18:2; 103-112.

40. Myrhaug H.T., Oxman A.D., Nilsen E., Johansen M., Oliver S. (2006) *Methods of consumer involvement in developing healthcare policy and research, clinical practice guidelines and patient information material.* Cochrane Database of Systematic Reviews, 2006/3

41. National Institute for Health Research (NIHR) (2012). *Patients and Public* [on-line] last accessed 18/06/2012 at URL http://www.crncc.nihr.ac.uk/PI

42. Nilsen E.S., Myrhaug H.T., Johansen M., Oliver S., Oxman A.D. (2009). *Methods of consumer involvement in developing healthcare policy and research, clinical practice guidelines and patient information material*. Cochrane Database of Systematic Reviews 2009/3

43. Oliver S, Milne R, Bradburn J, Buchanan P, Kerridge L, Walley T and Gabbay J. (2001). Investigating consumer perspectives on evaluating health technologies. *Evaluation*;7:468

44. Oliver SR, Rees RW, Clarke-Jones L, Milne R, Oakley AR, Gabbay J, et al. (2008). A multidimensional conceptual framework for analysing public involvement in health services research. Health Expectations; 11: 72-84

45. Patient-Centred Outcomes Research Institute. Patient-Centred Outcomes Research, University of Pittsburgh. (2013), http://www.pcori.org/research-we-support/pcor ; 2013

46. Peterson R.A, Albaum G., Kerin R.A. (1989). A note on alternative contact strategies in mail surveys. *Journal of the Market Research Society* 31:July; 409-418.

47. Popay J. Attree P. Hornby D. Milton B. Whitehead M. French B. Kowarzik U., Simpson N. Povall S. (2007). *Community engagement to address the wider social determinants of health: A review of evidence on impact, experience and process,* NICE, London. [on-line] last accessed 18/06/2012 at URL http://www.nice.org.uk/guidance/index.jsp?action=folder&o=34709

48. Richie J, Spencer L. (1994). *Qualitative data analysis for applied policy research.* In:A Bryman, RG Burgess (Eds). Analysis Qualitative Data, Routledge, London.

49. Sackman H. (1975). *Delphi Critique.* Lexington Books, Lexington, Mass.
50. Shield T., Campbell S., Rogers A. Worrall A., Chew-Graham C., Gask L. (2003). Quality indicators for mental health care in primary care. *Quality and Safety in Health Care* 12:2; 100–106.
51. Sitzia J, Wood N. (1998). Response rate in patient satisfaction research: an analysis of 210 published studies. *Int J Qual Health* Care; 10: 311–317.
52. Staley K, (2009). *Exploring impact: PI in NHS, public health and social care research'*. Eastleigh:INVOLVE.

For peer review only

53. Staniszewska S., Brett J., Mockford C., Barber R. (2011). The GRIPP checklist: strengthening the quality of patient and PI reporting in research. *International Journal of Technology Assessment in Health Care* 27:4; 391–399.

54. Walker A., Selfe J. (1996). The Delphi method: a useful tool for the allied health researcher. *British Journal of Therapy and Rehabilitation* 3:12, 677-681.

55. Wright D.N.M., Corner J.L., Hopkinson J.B., Foster C.L. (2007). The case for user involvement in research: the research priorities of cancer patients. *Breast Cancer Research* 9(Suppl 2): S3.

56. Wyatt K., Carter M., Mahtani V., Barnard A., Hawton A., Britten N. (2008). The impact of consumer involvement in research: an evaluation of consumer involvement in the London Primary Care Studies Programme. *Family Practice* 25; 154-161.

For peer review only

Exploring areas of consensus and conflict around values underpinning public involvement in health and social care research: A modified Delphi study

Journal:	*BMJ Open*
Manuscript ID:	bmjopen-2013-004217.R1
Article Type:	Research
Date Submitted by the Author:	09-Dec-2013
Complete List of Authors:	Snape, Dee; University of Liverpool, Public Health & Policy<br>Kirkham, Jamie; University of Liverpool, Biostatistics<br>Preston, Jennie; University of Liverpool, MRCN Community Liaison<br>Popay, Jennie; University of Lancaster, Division of Health Research<br>Britten, Nicky; University of Exeter, Institute of Health Service Research<br>Collins, Michelle; University of Lancaster, Division of Health Research<br>Froggatt, Katherine ; University of Lancaster, Division of Health Research<br>Gibson, Andy; University of Exeter, Institute of Health Service Research<br>Lobban, Fiona; University of Lancaster, Division of Health Research<br>Wyatt, Katrina; Peninsula College of Medicine and Dentistry,<br>Jacoby, Ann; University of Liverpool, Public Health & Policy
**Primary Subject Heading**:	Health services research
Secondary Subject Heading:	Research methods, Patient-centred medicine, Health policy, Evidence based practice, Ethics
Keywords:	Public involvement, Values, Conflict, Consensus, Health research, Delphi Technique

SCHOLARONE™
Manuscripts

For peer review only

Exploring areas of consensus and conflict around values underpinning public involvement in health and social care research: A modified Delphi study

Snape D [a], Kirkham J [b], Preston J [c], Popay J [d], Britten N [e], Collins, M [d], Froggatt K [d], Gibson A [e], Lobban F [d], Wyatt K [e], Jacoby A [a] *

a Department of Public Health and Policy, University of Liverpool, Liverpool, UK
b Department of Biostatistics, University of Liverpool, Liverpool, UK
c NIHR MRCN Consumer Liaison Manager, University of Liverpool, Liverpool, UK
d Division of Health Research, Lancaster University, Lancaster, UK
e Institute for Health Research, Exeter Medical School, Exeter, UK
* Corresponding author E-mail address ajacoby@liv.ac.uk

Address for correspondence:
Ann Jacoby
Department of Public Health and Policy
University of Liverpool
Whelan Building
The Quadrangle
Brownlow Hill
Liverpool
L69 3GB
UK

Email: ajacoby@liv.ac.uk
Tel: 00 44 151 794 5602
Fax: 00 44 151 794 5588

***Key words*:**
Public involvement
Values
Conflict
Consensus
Health research
Delphi technique

***Word count (excluding title page, abstract, references, tables & figures):* 5517**

For peer review only

ABSTRACT

Objective: There is growing interest in the potential benefits of public involvement (PI) in health and social care research. However, there has been little examination of values underpinning PI or how these values might differ for different groups with an interest in PI in the research process. We aimed to explore areas of consensus and conflict around normative, substantive and process-related values underpinning PI.

Design: Mixed method, three-phase, modified Delphi study, conducted as part of a larger multi-phase project.

Setting: The UK health and social care research community.

Participants: Stakeholders in public involvement in research, defined as: clinical and non-clinical academics, members of the public, research managers, commissioners and funders; identified via research networks, on-line searches and a literature review.

Results: We identified high levels of consensus for many normative, substantive and process-related issues. However there were also areas of conflict in relation to issues of bias and representativeness; and around whether the purpose of PI in health and social care research is to bring about service change or generate new knowledge. There were large differences by group in the percentages endorsing the ethical justification for PI; and the argument that PI equalises power imbalances. With regard to practical implementation of PI, research support infrastructures were reported as lacking. Participants reported shortcomings in the uptake and practice of PI. Embedding PI practice and evaluation in research study designs was seen as fundamental to strengthening the evidence base.

Conclusions: Our findings highlight the extent to which PI is already embedded in research. However they also highlight a need for 'best practice' standards to assist research teams to understand, implement and evaluate PI. These findings have been used in developing a Public Involvement Impact Assessment Framework (PiiAF), which offers guidance to researchers and members of the public involved in the PI process.

ARTICLE SUMMARY

Strengths and limitations of this study

- Despite growing interest In the UK and internationally in the potential benefits of public involvement (PI) in research, there has been little examination of the values underpinning it, or of how these values might be different for different stakeholders. As part of a large study developing guidance on how to assess the impact of public involvement in research we undertook a mixed method modified Delphi study which has provided primary evidence of areas of consensus and conflict around the value systems underpinning (PI) in research.

For peer review only

- Our mixed method, modified Delphi study involved a heterogeneous panel of PI experts, reflective of the range of key stakeholder groups and was geographically diverse.
- 'Consensus' thresholds were determined in advance of data collection; with two levels of consensus defined.
- A limitation of our study was that panellists likely were individuals with a strong commitment to PI; we can therefore say nothing about the values of those who choose not to engage with the PI enterprise. A further limitation is that response rates to the two-round survey were low and that our conclusions are potentially biased. However, a large percentage of the Round 1 survey responders also completed the Round 2 survey; and the quality of the data was high.
- Our findings provide important evidence about value consensus, but also highlight a need for 'best practice' standards to assist research teams in the PI process.

For peer review only

LAY SUMMARY

The purpose of this modified Delphi study was to examine areas of agreement and disagreement about the values underlying public involvement (PI) and the possible impacts of PI in health and social care research.

The study involved initial workshops with PI experts, to identify topics for exploration. Experts were representative of the various stakeholder groups subsequently recruited to a large-scale on-line survey. These were: members of the public involved in research; clinical and non-clinical researchers; research managers and research funders. Following the workshops, a pilot survey was conducted to develop and refine the survey questionnaires. Two survey rounds were then conducted, involving 318 respondents in round 1 and 231 in round 2. Both quantitative and qualitative responses were collected.

Overall, we identified high levels of agreement across the stakeholder groups for many of the issues explored. These areas of agreement reflect the extent to which PI is already embedded in health and social care research. However, we also found some areas of conflict between stakeholder groups.

Key findings in relation to the values underlying PI were -

- High levels of agreement that members of the public can and should influence how research is used
- Different and sometimes conflicting views across stakeholder groups about the possible biases and lack of representativeness in PI
- Different and sometimes conflicting views across stakeholder groups as to whether the purpose of PI in research is to bring about service change or generate new knowledge
- Agreement that there are tensions between what members of the public and researchers see as the purpose of research, and also what constitutes good research
- Differences between stakeholder groups in the percentages endorsing the ethical justification for PI

Our findings highlight a need for 'best practice' standards to assist researchers when involving members of the public in their research projects. These findings have been used in the development of Public Involvement Impact Assessment Framework (PiiAF), which offers guidance to both researchers and members of the public involved in the PI process.

For peer review only - http://bmjopen.bmj.com/site/about/guidelines.xhtml

1. INTRODUCTION

There is a growing interest internationally in the potential benefits of Public Involvement (PI) in health research[1-3]. Within the United Kingdom, the involvement of members of the public in research is firmly established in health and social care policy[4] and organisations including, for example, the National Institute for Health Research[5] increasingly identify public involvement (PI) as a pre-requisite for funding.

There is a plethora of literature illuminating the experiences of members of the public and professional researchers, and the process of PI and its potential impacts[6-9]. However, while studies report on use of PI in context of priority-setting[10, 11], the conduct of clinical trials[12] and the identification of treatment outcomes[13], there has been relatively little examination of the values underpinning PI, or about capturing and assessing the impact of PI effectively[14-18]. Possible reasons cited for the limited number of studies evaluating the impact of PI are: that evaluation is too difficult; and that PI is of intrinsic value and as such needs no further justification[16, 19-21]. The authors of the present paper acknowledge this latter perspective. However, we would argue that the current lack of a research evidence base around the impact of PI presents a challenge, since without such an evidence base it is difficult to ensure integrity of the PI endeavour, avoid potential adverse effects and ensure that it is adequately resourced. The modified Delphi study reported here was part of a larger MRC funded study (G0902155/93948) which aimed to review evidence on the values and impacts associated with PI, develop guidance on how these impacts can be assessed and contribute to the development of good practice standards for PI. The modified Delphi study utilised mixed methods to explore areas of consensus and conflict underpinning three over-arching value systems identified from a preceding literature review[22]. These values relate to (i) normative perspectives on PI, which consider involvement as an end in itself, for example, rights and empowerment; (ii) substantive perspectives which consider the consequences of PI, for example quality and relevance, and (iii) process-related values associated with good involvement, for example, partnership and equality.

2. AIM AND OBJECTIVES OF THE DELPHI STUDY

The overall aim of the modified Delphi Study was to explore areas of consensus and conflict around the normative, substantive and process-related values underpinning PI in health and social care research. The specific objectives were:

- To explore key issues identified from our literature review with PI experts
- To provide new knowledge about the views of different stakeholder on PI
- To contribute to the development of guidance on how to assess the impact of public involvement in research.

For peer review only - http://bmjopen.bmj.com/site/about/guidelines.xhtml

3. METHODS

In this study, we defined PI as an active partnership between members of the public and researchers in the research process, rather than the use of people as the 'subjects' of research. The term 'public' has been used as developed by the UK National Advisory Group INVOLVE[23], and includes patients and potential patients, carers and people who use health and social care services. Within the study reported here, PI has been achieved through the collaboration of academic researchers with experience of working in the field of PI, user investigators and the wider project's Public Advisory Group whose members contributed to all five phases of the work. Approval for the study was given by the University of Liverpool Research Ethics Committee. A mixed methods approach to data collection was employed in a three-stage, modified Delphi process:

i) Three Expert Workshops (participant total n=42) including members of the public, academic and user-researchers, researcher/clinicians, research funders and research managers were conducted to generate qualitative data. The range of normative, substantive and process-related values underpinning PI, identified in a previously conducted literature review[22] were discussed, to identify concepts to be explored at Round 1 (R1) and Round 2 (R2) of the subsequent modified Delphi survey;

ii) A pilot study to test the R1 survey questionnaire. As a strategy to reduce attrition[24, 25], careful attention was paid to the content and layout of the invitation e-mail, the survey layout and the clarity of questions. Piloting was conducted with academic (n=6) and user-researchers (n=3) and members of our Patient Advisory Group (n=2). Language, question type and questionnaire formatting were edited in response to participant feedback;

iii) A two-round modified Delphi survey was undertaken between November 2011 and September 2012 to explore areas of consensus and conflict around the normative, substantive and process-related values underpinning PI in health and social care research.

In this paper we describe the modified Delphi process and report on the findings relating to the values underpinning PI in health and social care research. A subsequent paper will report the survey findings in relation to perceived PI barriers and facilitators, perceived impacts of PI and potential approaches to its evaluation.

A modified Delphi technique

The main premise of the Delphi technique is the assumption that group opinion is more valid than individual opinion. The technique offers a reliable data collection method in circumstances where there is uncertainty or a paucity of knowledge surrounding the topic area under investigation[26-30]. While the Delphi technique has been used extensively within health and social science research[31-38], criticism of the technique includes the perception that it seeks to force consensus and so is weakened by not allowing panellists to elaborate on their views[32]. In response to this criticism, the current study used a modified Delphi technique which did not force consensus; rather panellists were provided with opportunities to

For peer review only

elaborate on why they held the views they expressed or endorsed[33], in order to allow us to try and tease out areas of conflict as well as areas of consensus.

There are a number of key concepts and assumptions which distinguish the basic Delphi technique from other research methods. These include anonymity, multi-stage iteration and controlled feedback, exploration of consensus via statistical group response and the use of experts[30, 39]. Each of these key characteristics was given due consideration as a means of enhancing the validity and reliability of the study design and the quality of responses[28, 40-43].

Sampling and the use of 'experts'

To reduce possible bias, our sampling strategy for panel composition was both reflective of the population subgroups under investigation and geographically diverse[31, 33]. To increase the reliability of the results a large heterogeneous panel of 'experts', consisting of distinct stakeholder groups was recruited[24]. We defined 'experts' as a group of *informed individuals*[28, 44, 45] – which, in this instance, was knowledge and/or experience of public involvement in health and social care research. Sampling was purposive. We aimed to capture the various PI perspectives and interests of members of the public, user, academic and clinical researchers, and research managers, directors, commissioners and funders. Potential panellists were identified in one of three ways: directly, through research team members' contacts and networks; through on-line searches of open-access research information and funding sites; and via a review of literature in the field of PI in health and social care research.

Anonymity

Complete anonymity could not be achieved as survey panellists and their responses were known to some members of the research team. However, anonymity between panellists was guaranteed. Reactions to panellists' opinions, key arguments and levels of consensus for each sub-group were fed back to panellists at R2 of the modified Delphi survey; each opinion carried the same weight and was afforded the same degree of importance in the analysis. In this way, subject bias was eliminated[33]. This approach also enabled panellists to be open and honest about their views on various issues as well as providing them with an equal opportunity to express an opinion without feeling pressured psychologically to conform to the views of others[33].

Quantitative data analysis

Determining how consensus cut-off points are reached and understanding how they are derived is often problematic, as the reporting of such criteria is limited within many published studies[29]. To address this, we sought statistical advice and defined specific criteria for determining consensus thresholds *a priori*.

For peer review only

We took, as representing **clear** consensus, endorsement or rejection of a statement by at least 60% of respondents. However, attention was also paid to the distribution of responses of the remaining 40%. Where these responses clustered in one response option only, consensus was not assumed and this item was further explored in R2 of the survey. We took, as representing **critical** consensus, endorsement or rejection of a statement by at least 70% of respondents. An additional criterion for critical consensus was that at least 55% of responses either endorsed or rejected the extreme categories in the scales.

At R2 the results from the R1 survey were fed back to panellists in the form of bar charts, textual summaries of statistical data and summarised descriptions of qualitative findings. Feedback included comparison of the responses of the different subgroups for each item. Particular attention was drawn to items demonstrating either within- or between-subgroup dissent. Items at R1 where between-subgroup differences were greater than 10% were further explored at R2. Also explored at R2 were any 'unexpected' (in the collective view of the study team) endorsement/rejection of items by the subgroups. We draw attention to these 'unexpected' responses in the findings section below.

Qualitative data analysis

In both R1 and R2, to enable in-depth exploration of the quantitative findings, panellists were provided with the opportunity to make further comments as they saw fit, through open questions. Thematic codes were identified using Framework Analysis[46], a matrix based method for ordering and synthesising data. The analysis was conducted by DS. Quality checking of the coding process and reduction of coding bias was ensured by AJ who reviewed 10% of the qualitative data. Firstly, data were reviewed inductively to identify recurring themes and concepts raised by panellists. These were coded and formed the initial major and sub themes; additional codes were then incorporated through an iterative process involving DS and AJ. The thematic framework was further refined before being applied systematically to the whole data set. This process facilitated the identification of any inconsistencies in coding which were subsequently discussed and reconciled.

4. RESULTS

The iterative nature of the Delphi technique enabled R2 of the survey to be informed by the results of R1. For this reason, the methods and results for both rounds are discussed together.

Panellists

At R1 seven hundred and forty (n=740) potential 'expert' panellists were invited, via e-mail, to participate in the on-line survey. Non-responders were e-mailed up to two reminder letters as appropriate, yielding a total response of three hundred and eighteen (n=318) (response rate 43%). Responding panellists self-selected themselves into one of five 'stakeholder' groups: clinical academic [CA], (20%); non-clinical academic [NCA], (28%);

For peer review only - http://bmjopen.bmj.com/site/about/guidelines.xhtml

member of the public [MP], (17%); research manager or funding / commissioning body employee [RM], (24%); or, occupying multiple roles [MR], (11%).

The characteristics of those who took part in R1 are shown in Table 1. Overall, participants had high levels of expertise, with most reporting having at least five years experience in research, and three-quarters having some responsibility for PI. However, there were also differences in level of expertise, members of the public being least likely to have lengthy research experience or any specific PPI responsibilities. In contrast, though fewer than half (n=134; 48%) of all panellists reported having had any formal training relevant to PI, members of the public were far more likely to have had such training (Table 1), while academic researchers tended to emphasise 'learned by doing'.

[INSERT TABLE 1]

Panellists reported being involved in a wide range of research roles including, for example: drafting research proposals and protocols (n=239; 86%); developing plain English summaries (n=238; 86%); sitting on project advisory and steering groups (n=228; 82%); presenting research findings (n=226; 82%); and being involved in data collection (n=223; 815). Panellists cited a number of practical learning opportunities which underpinned their PI skills acquisition; including, for example:

> *"Good networking".* [NCA, R1]
>
> *"…hard work building rapport and understanding of the patients' perspective, putting myself in their shoes".* [NCA, R1]
>
> *"Support from others, learning alongside others, and time".* [CA, R1]
>
> *"…so learning from mistakes and trying to listen to what both the patients and the researchers need to make it a meaningful experience".* [MP, R1]
>
> *"Various policy documents for engaging the public in research on which we can rest our local framework and priorities".* [RF, R1]

All panellists (n=318; response rate 43%) who submitted the R1 questionnaire were eligible to participate in R2. Of the 318 responders, three electronically 'opted out' of receiving further e-mail communications. The R2 questionnaire was therefore e-mailed to three hundred and fifteen (n=315) panellists. Up to two reminders were actioned as required. A total of two hundred and thirty one (n=231) R2 survey responses were received across the 5 stakeholder groups: clinical academic [CA], (17%); non-clinical academic [NCA], (29%); member of the public [MP], (18%); research manager or funding / commissioning body employee [RM], (24%); and, occupying multiple roles [MR], (12%); yielding a R2 response rate of 73% (of the 43% who took part in R1).

Arguments related to the nature of knowledge

We asked our panellists to comment on what kinds of knowledge they believed should inform health and social care research. Responses at R1 (see Figure 1) revealed critical consensus

For peer review only

across the different stakeholder groups (over 90% strongly agreed/agreed) for the substantive argument that members of the public have unique knowledge and expertise that is complementary to that of professionals/clinicians and researchers, and should be valued equally.

A comment from one clinical academic panellist illustrates this:

> *"It is important not to conflate the complementary perspectives of clinical / health, social care professional researchers versus methodologists. Both have important knowledge and expertise to bring to the table in informing health and social care research, as do patients, their informal carers and advocates and members of the public…it is important that all of these perspectives are regarded as equally important, rather than privileging any one view over the others".* [CA; R1]

Though it just failed to reach our definition of clear consensus, there was broad support across the stakeholder groups (58%) for the idea that while professionals/clinicians and researchers may also be service users, they cannot themselves represent user issues effectively. Since there was also some support across stakeholder groups (just over 50% strongly agreed/agreed) for the idea that both members of the research community and members of the public are likely to have biased views about research, we explored this issue of bias further in R2.

[Insert Figure 1]

At R2 we asked panellists to comment on whether they felt it mattered if different stakeholder groups held views which others considered biased. Forty three percent (43%) said "*yes*" they did feel it mattered, with a percentage range of 29%-59% across stakeholder groups. Individual stakeholder group responses are outlined in Table 2. [Insert Table 2]

A common theme across panellists' written comments and one articulated by a panellist holding multiple roles, was that all research actors are biased to some degree; holding different views based on knowledge and / or experience:

> *"All views will have a bias of some kind, but I think it is more important that the view is 'informed'. Bringing together the different perspectives in an open, collaborative way is one of the strengths of patient involvement, not a weakness".* [MR, R2].

Many panellists articulated that differences in perspective should not necessarily be viewed negatively; rather they should be acknowledged as a "*given*". Interestingly, at R2 the issue of power (who holds the power to enforce their viewpoint) was highlighted as more important than the question of bias.

Following from this, in R2 process values that involve *"building relationships of trust between the different stakeholder groups"* [NCA, R2], were frequently argued to be key for managing difference and essential for effective stakeholder partnerships and shared decision making.

For peer review only - http://bmjopen.bmj.com/site/about/guidelines.xhtml

Arguments related to the purposes of PI in health and social care research

There was critical consensus (over 80% strongly agreed/agreed) at R1, across all stakeholder groups, that members of the public should be involved in publicly funded research, and are entitled to say what and how research is undertaken (see Figure 2). There was also critical consensus that members of the public should be involved in research impacting on their own health or on NHS functioning; and clear consensus that they can influence how such research is used (see Figure 2). However, over 60% agreed/ strongly agreed that there are tensions between what members of the public and researchers see both as the purpose of research and what constitutes good research.

[insert Figure 2]

At R2 we asked panellists to comment on whether they believed these/such tensions within the arena of health and social research could be resolved. Given that tensions between the different stakeholder groups in the PI enterprise have been highlighted elsewhere [47], a somewhat unexpected finding from our data was critical consensus (75%) that tension resolution was possible. The percentage range across stakeholder groups was 67%-89%. Individual stakeholder group responses are outlined in Table 3.

[Insert Table 3]

Also unexpected was that, though tension was seen as an inevitable consequence of potential differences in perspective and/or expectations, it was not necessarily perceived as a negative issue. Rather it was seen by many as a means to stimulate critical debate:

> *"There should be tensions because that indicates passion; but the differences need to be managed in an open and transparent manner".* [MP, R2]
>
> *"Tensions are a natural consequence of different stakeholders having different priorities. To some extent the debate they lead tp could be regarded as conducive to better decisions.' [NCA, R2]*
>
> *"This is something to value, rather than to be concerned about! As long as those considering the various perspectives understand in advance that there may be differences in opinion, then there aren't 'tensions', just differences." [CA, R2]*
>
> *"…if researchers, clinicians and members of the public work together at the start of a trial, it will significantly reduce these tensions. However I also think that you can never entirely remove the tensions, and to some extent this is a good thing as the element of struggle helps to refine the research question."* [MP, R2]

Once again, the need to promote values of effective partnership working - including, for example, reciprocity, communication, reflexivity and learning from each other - was seen as key to tension resolution over time

There was support in panellists' written responses at R1 for the idea that PI training for members of the public needed to not only include an overview of research design and methods but should also involve education about political and policy context(s) which would lead to greater understanding of fundamental research drivers and processes. It was articulated by some panellists that training could also increase empowerment of members of

For peer review only - http://bmjopen.bmj.com/site/about/guidelines.xhtml

the public, promoting and facilitating partnership working within the research team. However, panel members also reflected that this should not stop academic researchers from recognising and valuing the experiential knowledge of members of the public themselves.

Arguments relating to the why and the how of PI

There was overall critical consensus (over 70% strongly agreed/agreed) that research is more ethical when members of the public are involved at all stages of the process. However, this statement was most strongly endorsed by members of the public (94% strongly agreed/agreed) and least endorsed by clinical (67%) and non-clinical (59%) academics. There was critical consensus across stakeholder groups (81% strongly agreed/agreed) that involvement is empowering for members of the public. Again, members of the public and those with multiple roles were much more likely to agree (75% strongly agreed/agreed) than were other groups (54%) that PI equalises the power between them and professionals. Clear consensus was also reached (over 65% agreed strongly/agreed) in relation to the statement that PI was often a "*tick the box*" exercise, with members of the public more likely to express disappointment at the lack of opportunity to influence the research process. Some panellists (across all stakeholder groups) suggested that involvement was still often seen as being tokenistic; with "*lip service*" only paid to the issue of PI:

> "*There is the perception that it is an inconvenient tick box exercise giving no added value and actually slowing research process down"* [CA, R2].
>
> *"Until researchers recognise the importance and value of public involvement, and how it can improve the quality of their research, they are likely to give it low priority"* [CA, R2].
>
> *"While PI is no longer optional it can still feel like a hassle for the academic community with gains that can seem remote. That needs to change otherwise people will continue being tokenistic*" [NCA, R2]

Panellists voiced a number of concerns about involving members of the public in the research process, including the lack of funding to involve the public in the early stages of research question and proposal development. Time pressures related to grant applications and project-specific time-lines were also highlighted as problematic, including lack of time to take account of public members' illness and/or work commitments, the lack of realistic timeframes for reviews and, not least, the lack of time needed to develop and nurture research team relationships.

At R1 a number of panellists expressed the view that a "*fit-for-purpose*" model of PI, which reflected study and social contexts, was a more appropriate way of assuring effective PI than the various "*levels of PI"* frameworks proposed in the PI literature[48]. Such frameworks were not popular with panellists, who argued that the hierarchical approach they embody to articulate different PI levels was misleading and not necessarily an indication of PI quality. The framework approach to PI was further criticised for not addressing all the facets of involvement, and as such having limited application, and becoming potentially a driver for

For peer review only - http://bmjopen.bmj.com/site/about/guidelines.xhtml

tokenism. Interestingly, INVOLVE[49] have recently updated their guidance to refer to 'approaches' rather than to 'levels' of involvement and also recognises that different approaches to PI might be used within the same project.

Following these responses we asked panellists at R2 whether, in their opinion, they felt there were any circumstances where PI was inappropriate. There was critical consensus among panellists that members of the public could be involved in all/any type of research - with the caveat that PI may be more challenging within the field of basic science research. The following observation made by one panellist, a research manager/ funder, serves to illustrate the potential for variation in the way members of the public can be involved:

> *"…hard to imagine a rationale for no involvement, but it will vary from project to project. One argument is often made that there shouldn't be public involvement in pure basic biomedical research and certainly the level of involvement will be very different to that, say, of a clinical trial. But there is still potential for involvement for example in wider public debate/involvement about evaluating this type of research and making policy decisions about how much money to invest in this type of research than more applied research. Some scientific projects may require human samples and there is a role for the public in the governance issues around this type of research - or motivating others to donate tissue etc. I have come across some aspects of clinical trials where, for example, two different scales for measuring depression were compared - a very technical and limited bit of work - with no room for influence from public involvement - but this was one part of a much larger trial where there was plenty of scope for involvement. So it may not always be appropriate for public involvement to influence the design and delivery of a project if it is very technical - but there is likely to be value of involvement in other aspects of the project around governance and funding decisions and perhaps dissemination in informing people that the research is going on - and explaining the implications of the research to a lay audience"* [RM, Round 2].

5. DISCUSSION

This paper reports findings from an on-line, modified Delphi study involving a range of stakeholder groups to explore normative, substantive and process-related values underpinning PI in health and social care research. This approach to gathering experts' opinions and suggestions without the need to bring them together physically, proved both versatile and resource-efficient. In this study, Delphi panellists' responses were fairly evenly balanced across the various stakeholder groups; and the response rate was, in our view, acceptable given the 'cold call' approach[50-52]. Allowing panellists an opportunity to comment on their interpretation of the items and to express their views via open-question feedback increased the reliability of the study and improved the validity of the results[42].

In the following discussion, we first consider key themes emerging from the data in relation to the normative, substantive and process-related values underpinning PI in health and social care research; second, we highlight the potential limitations of our research approach; and last, we present our conclusions.

For peer review only - http://bmjopen.bmj.com/site/about/guidelines.xhtml

Key themes

Our findings suggest high levels of self-reported expertise amongst members of the public involved in health and social care research, regardless of little 'formal' education and training in PI being reported. Panellists reported that timely and appropriate training is required by members of the public to support their involvement. Similarly, panellists also articulated that other actors in the research process require training in how to engage and support members of the public in their research roles. Support strategies suggested by panellists included, for example, advice and mentoring schemes.

Overall, our R1 survey identified critical consensus across stakeholder groups for many of the normative, substantive and process-related issues explored. These included: that members of the public have an entitlement to be involved in the research process, including in consideration of what research is undertaken and how it is used: and that different types of knowledge are important and that members of the public have unique knowledge. These areas of consensus highlight the extent to which PI is embedded in health and social care research. However, there were also areas of conflict by stakeholder group, for example there was wide variation in the percentages endorsing the normative position that research is more ethical where there is PI throughout. Although ethical concerns are paramount in the context of health and social care research, it is interesting to note that the two stakeholder groups least likely to endorse the importance of PI to ensure research probity were clinical and non-clinical academics. There was also disagreement about the extent to which PI was seen as equalising power between public and professionals; and about the substantive issues around potential stakeholder group bias and representativeness. These concerns about the lack of representativeness of members of the public who participate in PI in research initiatives have preoccupied the PI community for some considerable time[53, 54] and it has been argued that lack of clarity about these concepts has led to PI being devalued[53]. That it remains a focus of concern to our panellists highlights the need for a more sustained debate around the nature of 'representativeness' and about implementing involvement that is meaningful and appropriate.

Most health and social care research areas, including pre-clinical, were deemed appropriate for PI; with many panellists articulating their views about how and when it might be feasible to introduce and evaluate involvement. This reflects recent changes in commitment by UK funding bodies to incorporating PI across all research designs and at all stages of the research process[5]. Assessments about the appropriateness of PI need to be viewed within the overall opportunities for members of the public to influence project-specific agendas. A recent review of case studies conducted by Boote and colleagues[47] highlighted potential tensions between different stakeholder groups involved in PI. . However in our study the potential for tension was articulated as an inevitable consequence of collaborative working; individuals' agendas can be different when designing research studies. The acknowledgement and management of this standpoint was deemed essential in reconciling potential stakeholder difference. By promoting values of effective partnership including, for

For peer review only - http://bmjopen.bmj.com/site/about/guidelines.xhtml

example, communication, reflexivity and learning from each other, research relationships can develop overtime, and tension can lessen as individuals worked towards a common goal. The recognition that tensions are inevitable and can be used productively through questioning and discussion has also been highlighted by Abma and Widdershoven[55].

It is interesting to note that although panellists recognised the normative debates around involving members of the public in health and social care research and acknowledged significant progress in doing so, they also reported shortcomings in process and substantive-related issues around the practice of PI in research, not least of which is the need for quality standards wherein involvement is embedded methodologically in research study designs. Promoting and evaluating PI as 'normalised' research practice was seen as key to strengthening the evidence base around PI which in turn, it was argued, would reduce the practice of tokenism.

Delphi study limitations

In this study, we chose to adopt a modified Delphi approach for data collection. This involved the use of both fixed choice and open questions in order to try to maximise our understanding of the issues under consideration. As with all survey approaches, there are inevitably limitations to the depth of the data obtained; and it could be important to follow up key issues using more in-depth approaches, which would allow for more detailed exploration of less well understood and articulated issues.

Although Delphi techniques vary, face-to-face contact with participants at round one has been found useful in increasing the response rate[28]. However, due to the size of our sample, many of the panellists were targeted 'cold' without any prior notices. This approach may have had an impact on our response rate at R1. The use of reminders is generally endorsed in texts on survey methods[56] and in line with Peterson and colleagues[57], two reminder cover letters were e-mailed to non-responding participants at both R1 and R2 of the survey to stimulate additional responses.

That our response rate to Round 1 was low was unsurprising given our approach; however, it was encouraging that a large percentage of responders to Round 1 subsequently completed Round 2.The Delphi technique requires continued commitment from participants throughout the data collection process. Consideration must be given to the fact that individual time constraints and lack of familiarity with the Delphi technique may have prevented some participants from being able to make such a commitment. Nonetheless, the quality of the responses provided made clear that those who did take part were firmly committed to offering us detailed and extremely thoughtful answers to our questions.

It could be argued that another potential limitation relates to the representativeness of our panellists – though as discussed above, the issue of what 'representativeness' involves in the context of PI remains a thorny one. Under half of those we approached participated in Round 1 of the study, and this proportion was further reduced at Round 2. Since those who opted in to the survey self-selected the stakeholder group with which they aligned

For peer review only

themselves, we have no information about the groupings of those who opted out. We also have no information about other characteristics of interest for which we collected information from participants, for example, years of involvement in PI or extent of training in PI. We are therefore unable to comment meaningfully on the representativeness or otherwise of the study population. As previously clarified, we purposively set out to recruit PI experts to our study; it is likely, therefore, that those opting to take part were individuals with a particularly strong commitment to PI in research, who were therefore keen to endorse its validity. Conversely, our study may have failed to capture the views of people with experience, but no strong commitment to or opinions about PI. Our findings may therefore offer an overly optimistic picture. This needs to be borne in mind when interpreting the findings.

Conclusions

This study has identified a number of key issues around PI implementation, in relation to the different weightings given by different stakeholder groups to the values around PI; and the resultant potential for conflict. This study serves as one of the few attempts to empirically gauge the views of key stakeholders on these issues. The findings suggest that there is a large degree of consensus across many of these issues, but also a healthy level of debate in some important areas. While acknowledging the problem of representativeness of our panellists we feel that this Delphi survey provides us with important insight into the views of this group of PI experts.

Our findings also support the conclusion of Boote and colleagues[58] that there is a need for PI best practice standards which research teams can follow when seeking to involve members of the public in the research process. The aim of the wider MRC research within which this Delphi study sits was to develop and pilot guidance document to support assessment of the impacts of PI and to produce draft best practice standards. Findings from our modified Delphi study about commonly held PI values and about assessment of PI impacts (the latter is the subject of a separate paper) have contributed to this wider work by identifying areas where conflict is likely to arise and suggesting ways such conflict can be negotiated so the PI agenda can move forward meaningfully.

For peer review only

Figure legends

Figure 1: What kinds of knowledge should inform health and social care research?*

*Data taken from R1

Figure 2: What are the purposes of public involvement in health and social care research?*

*Data taken from R1

For peer review only

Table 1 Research experience by stakeholder group *

Stakeholder Group	n	Minimum 5 yrs research experience	Some PI responsibility	Formal training relevant to PI
Clinical Academic Researcher	63	52 (82.5%)	52 (82.5%)	27 (42.9%)
Non-Clinical Academic Researcher	88	70 (79.5%)	63 (71.6%)	27 (30.7%)
Member of the Public	55	33 (60.0%)	27 (49.1%)	35 (63.6%)
Research Manager/Funder	76	53 (69.7%)	64 (84.2%)	31 (40.8%)
Dual Role	34	30 (88.2%)	29 (85.3%)	14 (41.2%)

* Data taken from R1

Table 2: Does it matter if stakeholder groups hold views considered biased by others?*

Stakeholder Group	n	Bias Matters	Bias Doesn't Matter
Clinical Academic Researcher	39	23 (59%)	16 (41%)
Non-Clinical Academic Researcher	66	32 (48%)	34 (52%)
Member of the Public	41	17 (41%)	24 (59%)
Research Manager/Funder	56	16 (29%)	40 (71%)
Dual Role	27	10 (37%)	17 (63%)

* Data taken from R2

For peer review only

Table 3: Can tensions be resolved in health and social care research?*

Stakeholder Group	n	Tension Resolution YES	Tension Resolution NO
Clinical Academic Researcher	38	34 (89%)	4 (11%)
Non-Clinical Academic Researcher	66	44 (67%)	22 (33%)
Member of the Public	41	35 (85%)	6 (15%)
Research Manager/Funder	54	37 (69%)	17 (31%)
Dual Role	26	18 (69%)	8 (31%)

*Data taken from R2

For peer review only

Acknowledgements:

The authors of this paper are members of PiiAF research team, which involves researchers at the Universities of Lancaster, Exeter and Liverpool. Other members of the PiiAF team who contributed to the Delphi Study were: Felix Gradinger, Elaine Hewis, Tim Rawcliffe and Paula Williamson. Gradinger reviewed and commented on the survey questionnaires in light of the literature review he conducted as part of the wider MRC Study; Hewis and Rawcliffe took part in the expert workshops; Williamson contributed statistical expertise, including on the definition of consensus.

We would also like to acknowledge the valuable input of the members of the Public Advisory Group connected to this study: Bert Green, Faith Harris-Golesworthy, Dina Lew, Irene McGill, Nigel Pyart; and members of the Advisory Network. We are indebted to all those who took part in the Delphi study and wish to thank participants for their time and insight.

Funding statement:
The study was supported by the Medical Research Council's Methodology Research Programme [G0902155/93948]

Competing interests statement:

Professor Jennie Popay was a member of the MRC Methodology Research Programme at the time this grant was awarded, though had no involvement in the funding decision. There were no competing interests for the other authors.

Author contributions:

Snape was responsible for day-to-day management of the Delphi study, the qualitative data analysis and the drafting of the manuscript; Kirkham was responsible for management and analysis of the quantitative data; Preston assisted in the expert workshops, in development of the survey questionnaires and commented on the manuscript; Popay and Britten contributed to the conceptual development of the Delphi study and commented on the manuscript; Collins, Froggatt, Lobban and Wyatt all commented on the survey documents and the manuscript; Gibson assisted with the expert workshops, in development of the questionnaires and commented on the manuscript; Jacoby had responsibility for the overall conceptual and methodological development of the Delphi study, supervision of Snape, and drafting/finalising of the manuscript.

Data sharing statement:

We are exploring the scope for making the anonymised data files available from the corresponding author at the University of Liverpool, which would provide a permanent, citable and open access home for the dataset.

For peer review only

References

1. Patient-Centred Outcomes Research Institute (PCORI). Patient-Centred Outcomes Research, University of Pittsburgh. Last accessed 26/11/2013 at URL http://www.pcori.org/research-we-support/pcor

2. University of Western Australia. Consumer and Community Participation Policy. Last accessed 26/11/13 at URL http://www.sph.uwa.edu.au/

3. McKenzie A, Hanley B. Consumer and Community Participation in Health and Medical Research. A practical guide for health and medical research organisations. University of Western Australia and Telethon Institute for Child Health Research: Australia, 2009.

4. Department of Health. Research Governance Framework for Health and Social Care. 2nd Edition. London: Department of Health, 2005.

5. National Institute for Health Research (NIHR). Patients and Public. Last accessed 18/06/2012 at URL http://www.crncc.nihr.ac.uk/PI

6. Beresford P. User Involvement in research and evaluation: Liberation or Regulation? Social Policy & Society 2002;1(2):95-105.

7. Beresford P. User Involvement in research: Exploring the challenges. Nursing Times Research 2003;8(1):36-46.

8. Popay J, Attree P, Hornby D et al. Community engagement to address the wider social determinants of health: A review of evidence on impact, experience and process. London: NICE, 2007. Last accessed on 18/06/2012 at URL http://www.nice.org.uk/guidance/index.jsp?action=folder&o=34709

For peer review only

9. Wyatt K, Carter M, Mahtani V et al. The impact of consumer involvement in research: an evaluation of consumer involvement in the London Primary Care Studies Programme. Family Practice 2008;25(3):154-161.

10. Caron-Flinterman JF, Broerse JEW, Teerling J et al. Patients' priorities concerning health research: The case of asthma and COPD research in the Netherlands. Health Expect 2005;8(3):253-63.

11. Wright DNM, Corner JL, Hopkinson JB et al. The case for user involvement in research: the research priorities of cancer patients. Breast Cancer Research 2007;9(Suppl 2):S3.

12. Marsden J, Bradburn J. Patient and clinician collaboration in the design of a national randomised breast cancer trial. Health Expect 2004;7:6-17.

13. Hewlett S, de Wit M, Richards P et al. Patients and professionals as research partners: Challenges, practicalities, and benefits. Arthritis & Rheumatism 2006;55(4);676-680.

14. Minogue V, Boness J, Brown A et al. The impact of service user involvement in research. International Journal of Health Care Quality Assurance 2005;18(2):103-112.

15. Boote J, Telford R, Cooper C. Consumer involvement in health research: a review and research agenda. Health Policy 2002;61(2):213-236.

16. Staley K. Exploring impact: PI in NHS, public health and social care research. Eastleigh: INVOLVE, 2009.

For peer review only

17. Barber R, Boote JD, Parry GD et al.Can the impact of PI on research be evaluated? A mixed methods study. Health Expect 2011; 15(3):229-241.

18. Staniszewska S, Brett J, Mockford C et al. The GRIPP checklist: strengthening the quality of patient and PI reporting in research. International Journal of Technology Assessment in Health Care 2011;27(4):391–399.

19. Entwistle VA, Renfrew MJ, Yearley S et al. Lay perspectives: advantages for health researchers. BMJ 1998;316:463-466.

20. Oliver S, Milne R, Bradburn J et al. Investigating consumer perspectives on evaluating health technologies. Evaluation 2001;7:468.

21. Oliver SR, Rees RW, Clarke-Jones L et al. A multidimensional conceptual framework for analysing public involvement in health services research. Health Expect 2008;11:72-84.

22. Gradinger F, Britten N, Wyatt K et al. Values associated with Public Involvement in Health and Social Care Research. Health Expect, in press.

23. INVOLVE. INVOLVE Strategy 2012-2015. Putting people first in research. Eastleigh: INVOLVE 2012a. Last accessed 18/06/2012 at URL http://www.invo.org.uk

24. Duffield C. The Delphi Technique: a comparison of results obtained using two expert panels. International Journal of Nursing Studies 1993;30(3):227-237.

25. Hatton JM, Nunnelee JD. Research Priorities of Vascular Nursing. Journal of Vascular Nursing 1995;13(1):1-7.

26. Dalkey N, Helmer O. Delphi Technique: characteristics and sequence model to the use of experts. Management Science 1963;9(3):458-467.

For peer review only

27. Gallagher M, Hares T, Spencer J et al. The nominal group technique: a research tool for general practice. Family Practice 1993;10(1):76-81.

28. McKenna HP. The Delphi technique: a worthwhile approach for nursing? Journal of Advanced Nursing 1994;19(6);1221-1225.

29. Crisp J, Pelletier D, Duffield C et al. The Delphi Method? Nursing Research 1997;46(2):116-118.

30. Hassen F, Keeney S, McKenna H. Research Guidelines for the Delphi Survey Technique. Journal of Advanced Nursing 2000;32(4);1008-1015.

31. Jones J, Hunter D. Consensus methods for medical and health services research. BMJ 1995;311:276–380.

32. Walker A, Selfe J. The Delphi method: a useful tool for the allied health researcher. British Journal of Therapy and Rehabilitation 1996;3(12):677-681.

33. K

eeney S, Hassona F, McKennab HP. A critical review of the Delphi technique as a research methodology for nursing. International Journal of Nursing Studies 2001;38(2):195-200.

34. Campbell SM, Braspenning J, Hutchinson A et al. Research methods used in developing and applying quality indicators in primary care. Qual Saf Health 2002;11:358–364.

For peer review only

35. Daykin N, Sanidas M, Barley V et al. Developing consensus and interprofessional working in cancer services: the case of user involvement. Journal of Interprofessional Care 2002;16(4): 405–406.

36. Shield T, Campbell S, Rogers A et al. Quality indicators for mental health care in primary care. Qual Saf Health 2003;12(2);100–106.

37. Boote J, Barber R, Cooper C. Principles and indicators of successful consumer involvement in NHS research: Results of a Delphi study and subgroup analysis. Health Policy 2006;75(3):280-297.

38. Efstathiou N, Ameen J, Coll AM. A Delphi study to identify healthcare users' priorities for cancer care in Greece. European Journal of Oncology Nursing 2008;12(3):262–371.

39. Lynn MR, Layman EL, Englebardt SP. Nursing administration research priorities: a National Delphi Study. Journal of Nursing Administration 1998;28(5):7-11.

40. Li nstone HA, Turoff M. (eds). The Delphi Method: Techniques and Applications. Reading, Mass: Addison-Wesley, 1975.

41. Sackman H. Delphi Critique.). Lexington, Mass: Lexington Books, 1975.

42. McDonnell J, Meijler A, Kahan JP et al. Panellist consistency in the assessment of medical appropriateness. Health Policy 1996;37:139-152.

43. Hardy DJ, O'Brien AP, Gaskin CJ et al. Journal of Advanced Nursing 2004;46(1):95-109.

For peer review only

44. L

emmer B. Successive surveys of an expert panel: research in decision making with health visitors. Journal of Advanced Nursing 1998;27(3):538-545.

45. Green B, Jones M, Hughes D et al. Applying the Delphi technique in a study of GP's information requirement. Health and Social Care in the Community 1999;7(3):198-205.

46. Richie J, Spencer L. Qualitative data analysis for applied policy research. In: Bryman A, Burgess RG (Eds). Analysis Qualitative Data, London: Routledge, 1994, pp.173-194.

47. Boote J, Baird W, Beecroft C. Public involvement in the design stage of primary health research: A narrative review of case examples. Health Policy 2010; 95:10-23.

48. Hanley B, Bradburn J, Barnes M et al. Involving the public in NHS, public health and social care research: briefing notes for researchers, 2nd Edition. Eastleigh: INVOLVE, 2004.

49. INVOLVE. Briefing notes for researchers: involving the public in NHS, public health and social care research. Eastleigh: INVOLVE, 2012b Last accessed 8/07/2013 at URL http://www.invo.org.uk

50. Sitzia J, Wood N. Response rate in patient satisfaction research: an analysis of 210 published studies. Int J Qual Health Care, 1998;10:311–317.

51. Arber S. Designing samples. In: Gilbert N (ed). Researching Social Life. London: SAGE Publications, 2001.

52. Edwards P, Roberts I, Clarke M et al. Increasing response rates to postal questionnaires: systematic review. BMJ 2002;324:1183.

For peer review only

53. Beresford P, Campbell J. Disabled people, service users, user involvement and representation. Disability and Society 1994;9(3):315-25.

54. Morrow E, Boaz A, Brearley S et al. Handbook of service user involvement in nursing and health care research, Chpt 2, pp. 22-24. Chichester: Wiley Blackwell, 2012.

55. Abma TA, Widdershoven GAM. Sharing stories. Narrative and dialogue in responsive nursing evaluation. Evaluation and the Health Professions 2005;28:90-109.

56. McColl E, Jacoby A, Thomas L et al. Design and use of questionnaires: a review of best practice applicable to surveys of health service staff and patients. HTA 2001;5(31):101-132.

57. Peterson RA, Albaum G, Kerin RA. A note on alternative contact strategies in mail surveys. Journal of the Market Research Society 1989;31:409-418.

58. Boote J, Baird W, Sutton A. Public involvement in the systematic review process in health and social care: A narrative review of case examples. Health Policy 2011;102: 105-116.

For peer review only

Exploring areas of consensus and conflict around values underpinning public involvement in health and social care research: A modified Delphi study

Snape D [a] , Kirkham J [b] , Preston J [c], Popay J [d], Britten N [e], Collins, M [d], Froggatt K [d], Gibson A [e], Lobban F [d], Wyatt K [e], Jacoby A [a] *

a Department of Public Health and Policy, University of Liverpool, Liverpool, UK
b Department of Biostatistics, University of Liverpool, Liverpool, UK
c NIHR MRCN Consumer Liaison Manager, University of Liverpool, Liverpool, UK
d Division of Health Research, Lancaster University, Lancaster, UK
e Institute for Health Research, Exeter Medical School, Exeter, UK
* Corresponding author E-mail address ajacoby@liv.ac.uk

Address for correspondence:
Ann Jacoby
Department of Public Health and Policy
University of Liverpool
Whelan Building
The Quadrangle
Brownlow Hill
Liverpool
L69 3GB
UK

Email: ajacoby@liv.ac.uk
Tel: 00 44 151 794 5602
Fax: 00 44 151 794 5588

***Key words*:**
Public involvement
Values
Conflict
Consensus
Health research
Delphi technique

Word count (excluding title page, abstract, references, tables & figures): 5517

For peer review only

For peer review only - http://bmjopen.bmj.com/site/about/guidelines.xhtml

ABSTRACT

Objective: There is growing interest in the potential benefits of public involvement (PI) in health and social care research. However, there has been little examination of values underpinning PI or how these values might differ for different groups with an interest in PI in the research process. We aimed to explore areas of consensus and conflict around normative, substantive and process-related values underpinning PI.

Design: Mixed method, three-phase, modified Delphi study, conducted as part of a larger multi-phase project.

Setting: The UK health and social care research community.

Participants: Stakeholders in public involvement in research, defined as: clinical and non-clinical academics, members of the public, research managers, commissioners and funders; identified via research networks, on-line searches and a literature review.

Results: We identified high levels of consensus for many normative, substantive and process-related issues. However there were also areas of conflict in relation to issues of bias and representativeness; and around whether the purpose of PI in health and social care research is to bring about service change or generate new knowledge. There were large differences by group in the percentages endorsing the ethical justification for PI; and the argument that PI equalises power imbalances. With regard to practical implementation of PI, research support infrastructures were reported as lacking. Participants reported shortcomings in the uptake and practice of PI. Embedding PI practice and evaluation in research study designs was seen as fundamental to strengthening the evidence base.

Conclusions: Our findings highlight the extent to which PI is already embedded in research. However they also highlight a need for 'best practice' standards to assist research teams to understand, implement and evaluate PI. These findings have been used in developing a Public Involvement Impact Assessment Framework (PiiAF), which offers guidance to researchers and members of the public involved in the PI process.

ARTICLE SUMMARY

Strengths and limitations of this study

- Despite growing interest In the UK and internationally in the potential benefits of public involvement (PI) in research, there has been little examination of the values underpinning it, or of how these values might be different for different stakeholders. As part of a large study developing guidance on how to assess the impact of public involvement in research we undertook a mixed method modified Delphi study which has provided primary evidence of areas of consensus and conflict around the value systems underpinning (PI) in research.

For peer review only

- Our mixed method, modified Delphi study involved a heterogeneous panel of PI experts, reflective of the range of key stakeholder groups and was geographically diverse.
- 'Consensus' thresholds were determined in advance of data collection; with two levels of consensus defined.
- A limitation of our study was that panellists likely were individuals with a strong commitment to PI; we can therefore say nothing about the values of those who choose not to engage with the PI enterprise. A further limitation is that response rates to the two-round survey were low and that our conclusions are potentially biased. However, a large percentage of the Round 1 survey responders also completed the Round 2 survey; and the quality of the data was high.
- Our findings provide important evidence about value consensus, but also highlight a need for 'best practice' standards to assist research teams in the PI process.

For peer review only

LAY SUMMARY

The purpose of this modified Delphi study was to examine areas of agreement and disagreement about the values underlying public involvement (PI) and the possible impacts of PI in health and social care research.

The study involved initial workshops with PI experts, to identify topics for exploration. Experts were representative of the various stakeholder groups subsequently recruited to a large-scale on-line survey. These were: members of the public involved in research; clinical and non-clinical researchers; research managers and research funders. Following the workshops, a pilot survey was conducted to develop and refine the survey questionnaires. Two survey rounds were then conducted, involving 318 respondents in round 1 and 231 in round 2. Both quantitative and qualitative responses were collected.

Overall, we identified high levels of agreement across the stakeholder groups for many of the issues explored. These areas of agreement reflect the extent to which PI is already embedded in health and social care research. However, we also found some areas of conflict between stakeholder groups.

Key findings in relation to the values underlying PI were -

- High levels of agreement that members of the public can and should influence how research is used
- Different and sometimes conflicting views across stakeholder groups about the possible biases and lack of representativeness in PI
- Different and sometimes conflicting views across stakeholder groups as to whether the purpose of PI in research is to bring about service change or generate new knowledge
- Agreement that there are tensions between what members of the public and researchers see as the purpose of research, and also what constitutes good research
- Differences between stakeholder groups in the percentages endorsing the ethical justification for PI

Our findings highlight a need for 'best practice' standards to assist researchers when involving members of the public in their research projects. These findings have been used in the development of Public Involvement Impact Assessment Framework (PiiAF), which offers guidance to both researchers and members of the public involved in the PI process.

For peer review only - http://bmjopen.bmj.com/site/about/guidelines.xhtml

1. INTRODUCTION

There is a growing interest internationally in the potential benefits of Public Involvement (PI) in health research[1-3]. Within the United Kingdom, the involvement of members of the public in research is firmly established in health and social care policy[4] and organisations including, for example, the National Institute for Health Research[5] increasingly identify public involvement (PI) as a pre-requisite for funding.

There is a plethora of literature illuminating the experiences of members of the public and professional researchers, and the process of PI and its potential impacts[6-9]. However, while studies report on use of PI in context of priority-setting[10, 11], the conduct of clinical trials[12] and the identification of treatment outcomes[13], there has been relatively little examination of the values underpinning PI, or about capturing and assessing the impact of PI effectively[14-18]. Possible reasons cited for the limited number of studies evaluating the impact of PI are: that evaluation is too difficult; and that PI is of intrinsic value and as such needs no further justification[16, 19-21]. The authors of the present paper acknowledge this latter perspective. However, we would argue that the current lack of a research evidence base around the impact of PI presents a challenge, since without such an evidence base it is difficult to ensure integrity of the PI endeavour, avoid potential adverse effects and ensure that it is adequately resourced. The modified Delphi study reported here was part of a larger MRC funded study (G0902155/93948) which aimed to review evidence on the values and impacts associated with PI, develop guidance on how these impacts can be assessed and contribute to the development of good practice standards for PI. The modified Delphi study utilised mixed methods to explore areas of consensus and conflict underpinning three over-arching value systems identified from a preceding literature review[22]. These values relate to (i) normative perspectives on PI, which consider involvement as an end in itself, for example, rights and empowerment; (ii) substantive perspectives which consider the consequences of PI, for example quality and relevance, and (iii) process-related values associated with good involvement, for example, partnership and equality.

2. AIM AND OBJECTIVES OF THE DELPHI STUDY

The overall aim of the modified Delphi Study was to explore areas of consensus and conflict around the normative, substantive and process-related values underpinning PI in health and social care research. The specific objectives were:

- To explore key issues identified from our literature review with PI experts
- To provide new knowledge about the views of different stakeholder on PI
- To contribute to the development of guidance on how to assess the impact of public involvement in research.

For peer review only

3. METHODS

In this study, we defined PI as an active partnership between members of the public and researchers in the research process, rather than the use of people as the 'subjects' of research. The term 'public' has been used as developed by the UK National Advisory Group INVOLVE[23], and includes patients and potential patients, carers and people who use health and social care services. Within the study reported here, PI has been achieved through the collaboration of academic researchers with experience of working in the field of PI, user investigators and the wider project's Public Advisory Group whose members contributed to all five phases of the work. Approval for the study was given by the University of Liverpool Research Ethics Committee. A mixed methods approach to data collection was employed in a three-stage, modified Delphi process:

i) Three Expert Workshops (participant total n=42) including members of the public, academic and user-researchers, researcher/clinicians, research funders and research managers were conducted to generate qualitative data. The range of normative, substantive and process-related values underpinning PI, identified in a previously conducted literature review[22] were discussed, to identify concepts to be explored at Round 1 (R1) and Round 2 (R2) of the subsequent modified Delphi survey;

ii) A pilot study to test the R1 survey questionnaire. As a strategy to reduce attrition[24, 25], careful attention was paid to the content and layout of the invitation e-mail, the survey layout and the clarity of questions. Piloting was conducted with academic (n=6) and user-researchers (n=3) and members of our Patient Advisory Group (n=2). Language, question type and questionnaire formatting were edited in response to participant feedback;

iii) A two-round modified Delphi survey was undertaken between November 2011 and September 2012 to explore areas of consensus and conflict around the normative, substantive and process-related values underpinning PI in health and social care research.

In this paper we describe the modified Delphi process and report on the findings relating to the values underpinning PI in health and social care research. A subsequent paper will report the survey findings in relation to perceived PI barriers and facilitators, perceived impacts of PI and potential approaches to its evaluation.

A modified Delphi technique

The main premise of the Delphi technique is the assumption that group opinion is more valid than individual opinion. The technique offers a reliable data collection method in circumstances where there is uncertainty or a paucity of knowledge surrounding the topic area under investigation[26-30]. While the Delphi technique has been used extensively within health and social science research[31-38], criticism of the technique includes the perception that it seeks to force consensus and so is weakened by not allowing panellists to elaborate on their views[32]. In response to this criticism, the current study used a modified Delphi technique which did not force consensus; rather panellists were provided with opportunities to

For peer review only

elaborate on why they held the views they expressed or endorsed[33], in order to allow us to try and tease out areas of conflict as well as areas of consensus.

There are a number of key concepts and assumptions which distinguish the basic Delphi technique from other research methods. These include anonymity, multi-stage iteration and controlled feedback, exploration of consensus via statistical group response and the use of experts[30, 39]. Each of these key characteristics was given due consideration as a means of enhancing the validity and reliability of the study design and the quality of responses[28, 40-43].

Sampling and the use of 'experts'

To reduce possible bias, our sampling strategy for panel composition was both reflective of the population subgroups under investigation and geographically diverse[31, 33]. To increase the reliability of the results a large heterogeneous panel of 'experts', consisting of distinct stakeholder groups was recruited[24]. We defined 'experts' as a group of *informed individuals*[28, 44, 45] – which, in this instance, was knowledge and/or experience of public involvement in health and social care research. Sampling was purposive. We aimed to capture the various PI perspectives and interests of members of the public, user, academic and clinical researchers, and research managers, directors, commissioners and funders. Potential panellists were identified in one of three ways: directly, through research team members' contacts and networks; through on-line searches of open-access research information and funding sites; and via a review of literature in the field of PI in health and social care research.

Anonymity

Complete anonymity could not be achieved as survey panellists and their responses were known to some members of the research team. However, anonymity between panellists was guaranteed. Reactions to panellists' opinions, key arguments and levels of consensus for each sub-group were fed back to panellists at R2 of the modified Delphi survey; each opinion carried the same weight and was afforded the same degree of importance in the analysis. In this way, subject bias was eliminated[33]. This approach also enabled panellists to be open and honest about their views on various issues as well as providing them with an equal opportunity to express an opinion without feeling pressured psychologically to conform to the views of others[33].

Quantitative data analysis

Determining how consensus cut-off points are reached and understanding how they are derived is often problematic, as the reporting of such criteria is limited within many published studies[29]. To address this, we sought statistical advice and defined specific criteria for determining consensus thresholds *a priori*.

For peer review only - http://bmjopen.bmj.com/site/about/guidelines.xhtml

We took, as representing **clear** consensus, endorsement or rejection of a statement by at least 60% of respondents. However, attention was also paid to the distribution of responses of the remaining 40%. Where these responses clustered in one response option only, consensus was not assumed and this item was further explored in R2 of the survey. We took, as representing **critical** consensus, endorsement or rejection of a statement by at least 70% of respondents. An additional criterion for critical consensus was that at least 55% of responses either endorsed or rejected the extreme categories in the scales.

At R2 the results from the R1 survey were fed back to panellists in the form of bar charts, textual summaries of statistical data and summarised descriptions of qualitative findings. Feedback included comparison of the responses of the different subgroups for each item. Particular attention was drawn to items demonstrating either within- or between-subgroup dissent. Items at R1 where between-subgroup differences were greater than 10% were further explored at R2. Also explored at R2 were any 'unexpected' (in the collective view of the study team) endorsement/rejection of items by the subgroups. We draw attention to these 'unexpected' responses in the findings section below.

Qualitative data analysis

In both R1 and R2, to enable in-depth exploration of the quantitative findings, panellists were provided with the opportunity to make further comments as they saw fit, through open questions. Thematic codes were identified using Framework Analysis[46], a matrix based method for ordering and synthesising data. The analysis was conducted by DS. Quality checking of the coding process and reduction of coding bias was ensured by AJ who reviewed 10% of the qualitative data. Firstly, data were reviewed inductively to identify recurring themes and concepts raised by panellists. These were coded and formed the initial major and sub themes; additional codes were then incorporated through an iterative process involving DS and AJ. The thematic framework was further refined before being applied systematically to the whole data set. This process facilitated the identification of any inconsistencies in coding which were subsequently discussed and reconciled.

4. RESULTS

The iterative nature of the Delphi technique enabled R2 of the survey to be informed by the results of R1. For this reason, the methods and results for both rounds are discussed together.

Panellists

At R1 seven hundred and forty (n=740) potential 'expert' panellists were invited, via e-mail, to participate in the on-line survey. Non-responders were e-mailed up to two reminder letters as appropriate, yielding a total response of three hundred and eighteen (n=318) (response rate 43%). Responding panellists self-selected themselves into one of five 'stakeholder' groups: clinical academic [CA], (20%); non-clinical academic [NCA], (28%);

For peer review only

member of the public [MP], (17%); research manager or funding / commissioning body employee [RM], (24%); or, occupying multiple roles [MR], (11%).

The characteristics of those who took part in R1 are shown in Table 1. Overall, participants had high levels of expertise, with most reporting having at least five years experience in research, and three-quarters having some responsibility for PI. However, there were also differences in level of expertise, members of the public being least likely to have lengthy research experience or any specific PPI responsibilities. In contrast, though fewer than half (n=134; 48%) of all panellists reported having had any formal training relevant to PI, members of the public were far more likely to have had such training (Table 1), while academic researchers tended to emphasise 'learned by doing'.

[INSERT TABLE 1]

Panellists reported being involved in a wide range of research roles including, for example: drafting research proposals and protocols (n=239; 86%); developing plain English summaries (n=238; 86%); sitting on project advisory and steering groups (n=228; 82%); presenting research findings (n=226; 82%); and being involved in data collection (n=223; 815). Panellists cited a number of practical learning opportunities which underpinned their PI skills acquisition; including, for example:

> *"Good networking".* [NCA, R1]

> *"…hard work building rapport and understanding of the patients' perspective, putting myself in their shoes".* [NCA, R1]

> *"Support from others, learning alongside others, and time".* [CA, R1]

> *"…so learning from mistakes and trying to listen to what both the patients and the researchers need to make it a meaningful experience".* [MP, R1]

> *"Various policy documents for engaging the public in research on which we can rest our local framework and priorities".* [RF, R1]

All panellists (n=318; response rate 43%) who submitted the R1 questionnaire were eligible to participate in R2. Of the 318 responders, three electronically 'opted out' of receiving further e-mail communications. The R2 questionnaire was therefore e-mailed to three hundred and fifteen (n=315) panellists. Up to two reminders were actioned as required. A total of two hundred and thirty one (n=231) R2 survey responses were received across the 5 stakeholder groups: clinical academic [CA], (17%); non-clinical academic [NCA], (29%); member of the public [MP], (18%); research manager or funding / commissioning body employee [RM], (24%); and, occupying multiple roles [MR], (12%); yielding a R2 response rate of 73% (of the 43% who took part in R1).

Arguments related to the nature of knowledge

We asked our panellists to comment on what kinds of knowledge they believed should inform health and social care research. Responses at R1 (see Figure 1) revealed critical consensus

For peer review only

across the different stakeholder groups (over 90% strongly agreed/agreed) for the substantive argument that members of the public have unique knowledge and expertise that is complementary to that of professionals/clinicians and researchers, and should be valued equally.

A comment from one clinical academic panellist illustrates this:

> *"It is important not to conflate the complementary perspectives of clinical / health, social care professional researchers versus methodologists. Both have important knowledge and expertise to bring to the table in informing health and social care research, as do patients, their informal carers and advocates and members of the public…it is important that all of these perspectives are regarded as equally important, rather than privileging any one view over the others".* [CA; R1]

Though it just failed to reach our definition of clear consensus, there was broad support across the stakeholder groups (58%) for the idea that while professionals/clinicians and researchers may also be service users, they cannot themselves represent user issues effectively. Since there was also some support across stakeholder groups (just over 50% strongly agreed/agreed) for the idea that both members of the research community and members of the public are likely to have biased views about research, we explored this issue of bias further in R2.

[Insert Figure 1]

At R2 we asked panellists to comment on whether they felt it mattered if different stakeholder groups held views which others considered biased. Forty three percent (43%) said "*yes"* they did feel it mattered, with a percentage range of 29%-59% across stakeholder groups. Individual stakeholder group responses are outlined in Table 2. [Insert Table 2]

A common theme across panellists' written comments and one articulated by a panellist holding multiple roles, was that all research actors are biased to some degree; holding different views based on knowledge and / or experience:

> *"All views will have a bias of some kind, but I think it is more important that the view is 'informed'. Bringing together the different perspectives in an open, collaborative way is one of the strengths of patient involvement, not a weakness".* [MR, R2].

Many panellists articulated that differences in perspective should not necessarily be viewed negatively; rather they should be acknowledged as a "*given***"**. Interestingly, at R2 the issue of power (who holds the power to enforce their viewpoint) was highlighted as more important than the question of bias.

Following from this, in R2 process values that involve *"building relationships of trust between the different stakeholder groups"* [NCA, R2], were frequently argued to be key for managing difference and essential for effective stakeholder partnerships and shared decision making.

For peer review only

Arguments related to the purposes of PI in health and social care research

There was critical consensus (over 80% strongly agreed/agreed) at R1, across all stakeholder groups, that members of the public should be involved in publicly funded research, and are entitled to say what and how research is undertaken (see Figure 2). There was also critical consensus that members of the public should be involved in research impacting on their own health or on NHS functioning; and clear consensus that they can influence how such research is used (see Figure 2). However, over 60% agreed/ strongly agreed that there are tensions between what members of the public and researchers see both as the purpose of research and what constitutes good research.

[insert Figure 2]

At R2 we asked panellists to comment on whether they believed these/such tensions within the arena of health and social research could be resolved. Given that tensions between the different stakeholder groups in the PI enterprise have been highlighted elsewhere [47], a somewhat unexpected finding from our data was critical consensus (75%) that tension resolution was possible. The percentage range across stakeholder groups was 67%-89%. Individual stakeholder group responses are outlined in Table 3.

[Insert Table 3]

Also unexpected was that, though tension was seen as an inevitable consequence of potential differences in perspective and/or expectations, it was not necessarily perceived as a negative issue. Rather it was seen by many as a means to stimulate critical debate:

> *"There should be tensions because that indicates passion; but the differences need to be managed in an open and transparent manner".* [MP, R2]
>
> *"Tensions are a natural consequence of different stakeholders having different priorities. To some extent the debate they lead tp could be regarded as conducive to better decisions.' [NCA, R2]*
>
> *"This is something to value, rather than to be concerned about! As long as those considering the various perspectives understand in advance that there may be differences in opinion, then there aren't 'tensions', just differences." [CA, R2]*
>
> *"…if researchers, clinicians and members of the public work together at the start of a trial, it will significantly reduce these tensions. However I also think that you can never entirely remove the tensions, and to some extent this is a good thing as the element of struggle helps to refine the research question."* [MP, R2]

Once again, the need to promote values of effective partnership working - including, for example, reciprocity, communication, reflexivity and learning from each other - was seen as key to tension resolution over time

There was support in panellists' written responses at R1 for the idea that PI training for members of the public needed to not only include an overview of research design and methods but should also involve education about political and policy context(s) which would lead to greater understanding of fundamental research drivers and processes. It was articulated by some panellists that training could also increase empowerment of members of

For peer review only - http://bmjopen.bmj.com/site/about/guidelines.xhtml

the public, promoting and facilitating partnership working within the research team. However, panel members also reflected that this should not stop academic researchers from recognising and valuing the experiential knowledge of members of the public themselves.

Arguments relating to the why and the how of PI

There was overall critical consensus (over 70% strongly agreed/agreed) that research is more ethical when members of the public are involved at all stages of the process. However, this statement was most strongly endorsed by members of the public (94% strongly agreed/agreed) and least endorsed by clinical (67%) and non-clinical (59%) academics. There was critical consensus across stakeholder groups (81% strongly agreed/agreed) that involvement is empowering for members of the public. Again, members of the public and those with multiple roles were much more likely to agree (75% strongly agreed/agreed) than were other groups (54%) that PI equalises the power between them and professionals. Clear consensus was also reached (over 65% agreed strongly/agreed) in relation to the statement that PI was often a "*tick the box*" exercise, with members of the public more likely to express disappointment at the lack of opportunity to influence the research process. Some panellists (across all stakeholder groups) suggested that involvement was still often seen as being tokenistic; with "*lip service*" only paid to the issue of PI:

> "*There is the perception that it is an inconvenient tick box exercise giving no added value and actually slowing research process down"* [CA, R2].
>
> *"Until researchers recognise the importance and value of public involvement, and how it can improve the quality of their research, they are likely to give it low priority"* [CA, R2].
>
> *"While PI is no longer optional it can still feel like a hassle for the academic community with gains that can seem remote. That needs to change otherwise people will continue being tokenistic*" [NCA, R2]

Panellists voiced a number of concerns about involving members of the public in the research process, including the lack of funding to involve the public in the early stages of research question and proposal development. Time pressures related to grant applications and project-specific time-lines were also highlighted as problematic, including lack of time to take account of public members' illness and/or work commitments, the lack of realistic timeframes for reviews and, not least, the lack of time needed to develop and nurture research team relationships.

At R1 a number of panellists expressed the view that a "*fit-for-purpose*" model of PI, which reflected study and social contexts, was a more appropriate way of assuring effective PI than the various "*levels of PI"* frameworks proposed in the PI literature[48]. Such frameworks were not popular with panellists, who argued that the hierarchical approach they embody to articulate different PI levels was misleading and not necessarily an indication of PI quality. The framework approach to PI was further criticised for not addressing all the facets of involvement, and as such having limited application, and becoming potentially a driver for

For peer review only - http://bmjopen.bmj.com/site/about/guidelines.xhtml

tokenism. Interestingly, INVOLVE[49] have recently updated their guidance to refer to ‘approaches’ rather than to ‘levels’ of involvement and also recognises that different approaches to PI might be used within the same project.

Following these responses we asked panellists at R2 whether, in their opinion, they felt there were any circumstances where PI was inappropriate. There was critical consensus among panellists that members of the public could be involved in all/any type of research - with the caveat that PI may be more challenging within the field of basic science research. The following observation made by one panellist, a research manager/ funder, serves to illustrate the potential for variation in the way members of the public can be involved:

> *“…hard to imagine a rationale for no involvement, but it will vary from project to project. One argument is often made that there shouldn't be public involvement in pure basic biomedical research and certainly the level of involvement will be very different to that, say, of a clinical trial. But there is still potential for involvement for example in wider public debate/involvement about evaluating this type of research and making policy decisions about how much money to invest in this type of research than more applied research. Some scientific projects may require human samples and there is a role for the public in the governance issues around this type of research - or motivating others to donate tissue etc. I have come across some aspects of clinical trials where, for example, two different scales for measuring depression were compared - a very technical and limited bit of work - with no room for influence from public involvement - but this was one part of a much larger trial where there was plenty of scope for involvement. So it may not always be appropriate for public involvement to influence the design and delivery of a project if it is very technical - but there is likely to be value of involvement in other aspects of the project around governance and funding decisions and perhaps dissemination in informing people that the research is going on - and explaining the implications of the research to a lay audience”* [RM, Round 2].

5. DISCUSSION

This paper reports findings from an on-line, modified Delphi study involving a range of stakeholder groups to explore normative, substantive and process-related values underpinning PI in health and social care research. This approach to gathering experts’ opinions and suggestions without the need to bring them together physically, proved both versatile and resource-efficient. In this study, Delphi panellists’ responses were fairly evenly balanced across the various stakeholder groups; and the response rate was, in our view, acceptable given the ‘cold call’ approach[50-52]. Allowing panellists an opportunity to comment on their interpretation of the items and to express their views via open-question feedback increased the reliability of the study and improved the validity of the results[42].

In the following discussion, we first consider key themes emerging from the data in relation to the normative, substantive and process-related values underpinning PI in health and social care research; second, we highlight the potential limitations of our research approach; and last, we present our conclusions.

For peer review only

Key themes

Our findings suggest high levels of self-reported expertise amongst members of the public involved in health and social care research, regardless of little ‘formal’ education and training in PI being reported. Panellists reported that timely and appropriate training is required by members of the public to support their involvement. Similarly, panellists also articulated that other actors in the research process require training in how to engage and support members of the public in their research roles. Support strategies suggested by panellists included, for example, advice and mentoring schemes.

Overall, our R1 survey identified critical consensus across stakeholder groups for many of the normative, substantive and process-related issues explored. These included: that members of the public have an entitlement to be involved in the research process, including in consideration of what research is undertaken and how it is used: and that different types of knowledge are important and that members of the public have unique knowledge. These areas of consensus highlight the extent to which PI is embedded in health and social care research. However, there were also areas of conflict by stakeholder group, for example there was wide variation in the percentages endorsing the normative position that research is more ethical where there is PI throughout. Although ethical concerns are paramount in the context of health and social care research, it is interesting to note that the two stakeholder groups least likely to endorse the importance of PI to ensure research probity were clinical and non-clinical academics. There was also disagreement about the extent to which PI was seen as equalising power between public and professionals; and about the substantive issues around potential stakeholder group bias and representativeness. These concerns about the lack of representativeness of members of the public who participate in PI in research initiatives have preoccupied the PI community for some considerable time[53, 54] and it has been argued that lack of clarity about these concepts has led to PI being devalued[53]. That it remains a focus of concern to our panellists highlights the need for a more sustained debate around the nature of ‘representativeness’ and about implementing involvement that is meaningful and appropriate.

Most health and social care research areas, including pre-clinical, were deemed appropriate for PI; with many panellists articulating their views about how and when it might be feasible to introduce and evaluate involvement. This reflects recent changes in commitment by UK funding bodies to incorporating PI across all research designs and at all stages of the research process[5]. Assessments about the appropriateness of PI need to be viewed within the overall opportunities for members of the public to influence project-specific agendas. A recent review of case studies conducted by Boote and colleagues[47] highlighted potential tensions between different stakeholder groups involved in PI. . However in our study the potential for tension was articulated as an inevitable consequence of collaborative working; individuals’ agendas can be different when designing research studies. The acknowledgement and management of this standpoint was deemed essential in reconciling potential stakeholder difference. By promoting values of effective partnership including, for

For peer review only - http://bmjopen.bmj.com/site/about/guidelines.xhtml

example, communication, reflexivity and learning from each other, research relationships can develop overtime, and tension can lessen as individuals worked towards a common goal. The recognition that tensions are inevitable and can be used productively through questioning and discussion has also been highlighted by Abma and Widdershoven[55].

It is interesting to note that although panellists recognised the normative debates around involving members of the public in health and social care research and acknowledged significant progress in doing so, they also reported shortcomings in process and substantive-related issues around the practice of PI in research, not least of which is the need for quality standards wherein involvement is embedded methodologically in research study designs. Promoting and evaluating PI as 'normalised' research practice was seen as key to strengthening the evidence base around PI which in turn, it was argued, would reduce the practice of tokenism.

Delphi study limitations

In this study, we chose to adopt a modified Delphi approach for data collection. This involved the use of both fixed choice and open questions in order to try to maximise our understanding of the issues under consideration. As with all survey approaches, there are inevitably limitations to the depth of the data obtained; and it could be important to follow up key issues using more in-depth approaches, which would allow for more detailed exploration of less well understood and articulated issues.

Although Delphi techniques vary, face-to-face contact with participants at round one has been found useful in increasing the response rate[28]. However, due to the size of our sample, many of the panellists were targeted 'cold' without any prior notices. This approach may have had an impact on our response rate at R1. The use of reminders is generally endorsed in texts on survey methods[56] and in line with Peterson and colleagues[57], two reminder cover letters were e-mailed to non-responding participants at both R1 and R2 of the survey to stimulate additional responses.

That our response rate to Round 1 was low was unsurprising given our approach; however, it was encouraging that a large percentage of responders to Round 1 subsequently completed Round 2.The Delphi technique requires continued commitment from participants throughout the data collection process. Consideration must be given to the fact that individual time constraints and lack of familiarity with the Delphi technique may have prevented some participants from being able to make such a commitment. Nonetheless, the quality of the responses provided made clear that those who did take part were firmly committed to offering us detailed and extremely thoughtful answers to our questions.

It could be argued that another potential limitation relates to the representativeness of our panellists – though as discussed above, the issue of what 'representativeness' involves in the context of PI remains a thorny one. Under half of those we approached participated in Round 1 of the study, and this proportion was further reduced at Round 2. Since those who opted in to the survey self-selected the stakeholder group with which they aligned

For peer review only - http://bmjopen.bmj.com/site/about/guidelines.xhtml

themselves, we have no information about the groupings of those who opted out. We also have no information about other characteristics of interest for which we collected information from participants, for example, years of involvement in PI or extent of training in PI. We are therefore unable to comment meaningfully on the representativeness or otherwise of the study population. As previously clarified, we purposively set out to recruit PI experts to our study; it is likely, therefore, that those opting to take part were individuals with a particularly strong commitment to PI in research, who were therefore keen to endorse its validity. Conversely, our study may have failed to capture the views of people with experience, but no strong commitment to or opinions about PI. Our findings may therefore offer an overly optimistic picture. This needs to be borne in mind when interpreting the findings.

Conclusions

This study has identified a number of key issues around PI implementation, in relation to the different weightings given by different stakeholder groups to the values around PI; and the resultant potential for conflict. This study serves as one of the few attempts to empirically gauge the views of key stakeholders on these issues. The findings suggest that there is a large degree of consensus across many of these issues, but also a healthy level of debate in some important areas. While acknowledging the problem of representativeness of our panellists we feel that this Delphi survey provides us with important insight into the views of this group of PI experts.

Our findings also support the conclusion of Boote and colleagues[58] that there is a need for PI best practice standards which research teams can follow when seeking to involve members of the public in the research process. The aim of the wider MRC research within which this Delphi study sits was to develop and pilot guidance document to support assessment of the impacts of PI and to produce draft best practice standards. Findings from our modified Delphi study about commonly held PI values and about assessment of PI impacts (the latter is the subject of a separate paper) have contributed to this wider work by identifying areas where conflict is likely to arise and suggesting ways such conflict can be negotiated so the PI agenda can move forward meaningfully.

For peer review only

Acknowledgements:

The authors of this paper are members of PiiAF research team, which involves researchers at the Universities of Lancaster, Exeter and Liverpool. Other members of the PiiAF team who contributed to the Delphi Study were: Felix Gradinger, Elaine Hewis, Tim Rawcliffe and Paula Williamson. Gradinger reviewed and commented on the survey questionnaires in light of the literature review he conducted as part of the wider MRC Study; Hewis and Rawcliffe took part in the expert workshops; Williamson contributed statistical expertise, including on the definition of consensus.

We would also like to acknowledge the valuable input of the members of the Public Advisory Group connected to this study: Bert Green, Faith Harris-Golesworthy, Dina Lew, Irene McGill, Nigel Pyart; and members of the Advisory Network. We are indebted to all those who took part in the Delphi study and wish to thank participants for their time and insight.

Funding statement:
The study was supported by the Medical Research Council's Methodology Research Programme [G0902155/93948]

Competing interests statement:

Professor Jennie Popay was a member of the MRC Methodology Research Programme at the time this grant was awarded, though had no involvement in the funding decision. There were no competing interests for the other authors.

Author contributions:

Snape was responsible for day-to-day management of the Delphi study, the qualitative data analysis and the drafting of the manuscript; Kirkham was responsible for management and analysis of the quantitative data; Preston assisted in the expert workshops, in development of the survey questionnaires and commented on the manuscript; Popay and Britten contributed to the conceptual development of the Delphi study and commented on the manuscript; Collins, Froggatt, Lobban and Wyatt all commented on the survey documents and the manuscript; Gibson assisted with the expert workshops, in development of the questionnaires and commented on the manuscript; Jacoby had responsibility for the overall conceptual and methodological development of the Delphi study, supervision of Snape, and drafting/finalising of the manuscript.

Data sharing statement:

We are exploring the scope for making the anonymised data files available from the corresponding author at the University of Liverpool, which would provide a permanent, citable and open access home for the dataset.

For peer review only

Table 1 Research experience by stakeholder group *

Stakeholder Group	n	Minimum 5 yrs research experience	Some PI responsibility	Formal training relevant to PI
Clinical Academic Researcher	63	52 (82.5%)	52 (82.5%)	27 (42.9%)
Non-Clinical Academic Researcher	88	70 (79.5%)	63 (71.6%)	27 (30.7%)
Member of the Public	55	33 (60.0%)	27 (49.1%)	35 (63.6%)
Research Manager/Funder	76	53 (69.7%)	64 (84.2%)	31 (40.8%)
Dual Role	34	30 (88.2%)	29 (85.3%)	14 (41.2%)

* Data taken from R1

For peer review only

Figure 1: What kinds of knowledge should inform health and social care research?*

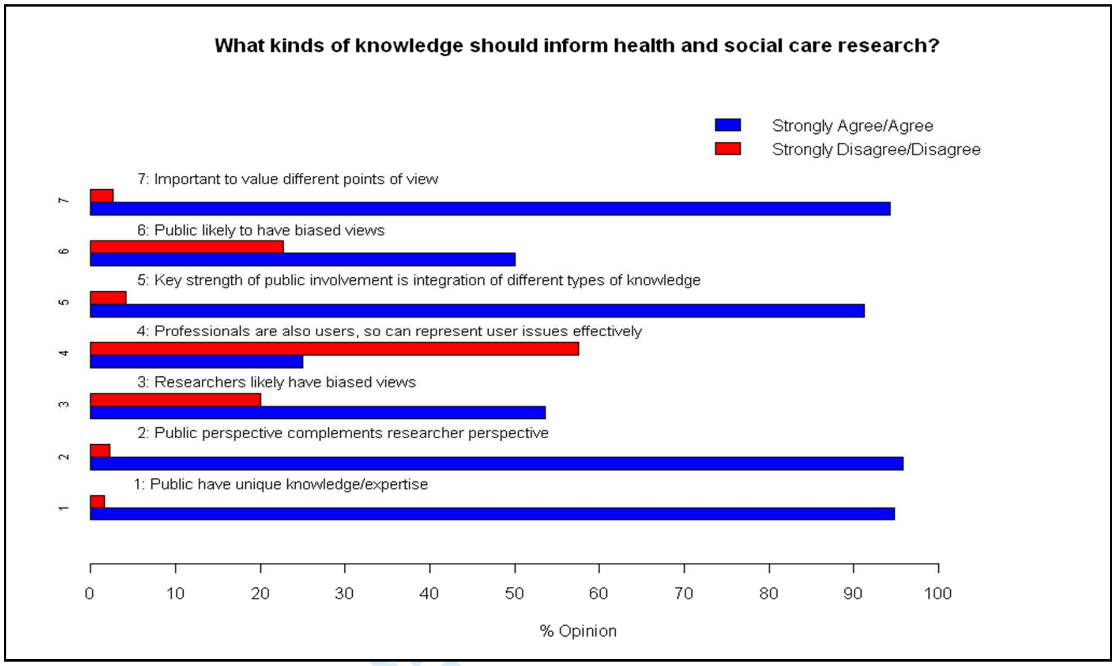

*Data taken from R1

For peer review only

Figure 2: What are the purposes of public involvement in health and social care research?*

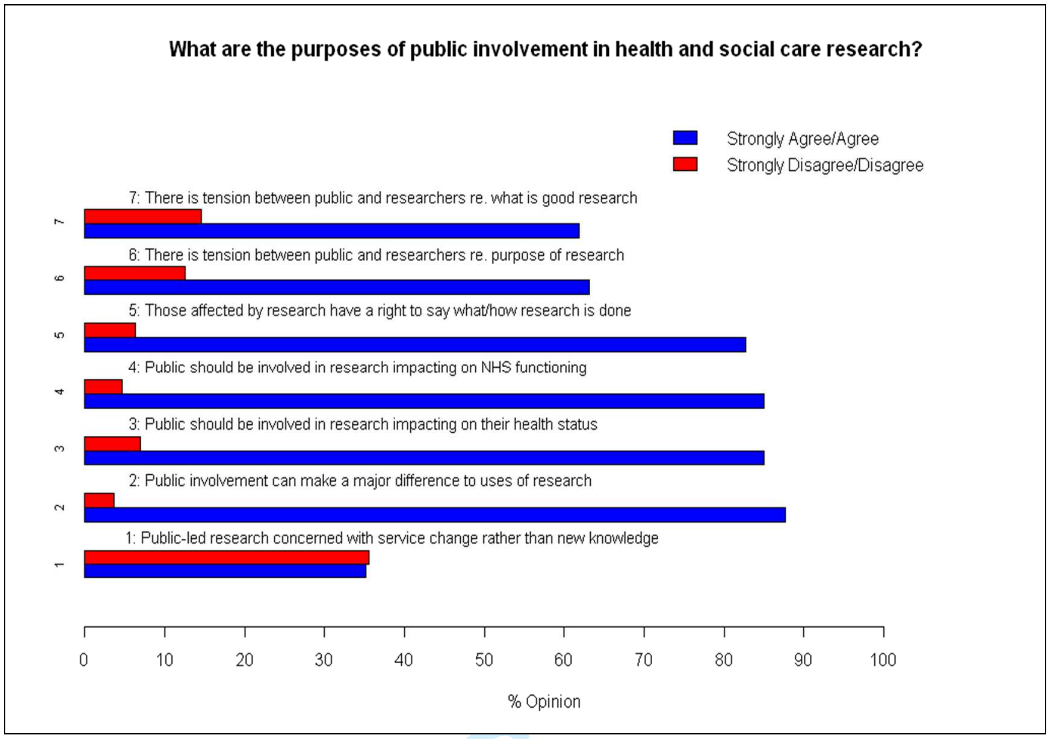

*Data taken from R1

Table 2: Does it matter if stakeholder groups hold views considered biased by others?*

Stakeholder Group	n	Bias Matters	Bias Doesn't Matter
Clinical Academic Researcher	39	23 (59%)	16 (41%)
Non-Clinical Academic Researcher	66	32 (48%)	34 (52%)
Member of the Public	41	17 (41%)	24 (59%)
Research Manager/Funder	56	16 (29%)	40 (71%)
Dual Role	27	10 (37%)	17 (63%)

* Data taken from R2

For peer review only

Table 3: Can tensions be resolved in health and social care research?*

Stakeholder Group	n	Tension Resolution YES	Tension Resolution NO
Clinical Academic Researcher	38	34 (89%)	4 (11%)
Non-Clinical Academic Researcher	66	44 (67%)	22 (33%)
Member of the Public	41	35 (85%)	6 (15%)
Research Manager/Funder	54	37 (69%)	17 (31%)
Dual Role	26	18 (69%)	8 (31%)

*Data taken from R2

For peer review only

References

1. Patient-Centred Outcomes Research Institute (PCORI). Patient-Centred Outcomes Research, University of Pittsburgh. Last accessed 26/11/2013 at URL http://www.pcori.org/research-we-support/pcor

2. University of Western Australia. Consumer and Community Participation Policy. Last accessed 26/11/13 at URL http://www.sph.uwa.edu.au/

3. McKenzie A, Hanley B. Consumer and Community Participation in Health and Medical Research. A practical guide for health and medical research organisations. University of Western Australia and Telethon Institute for Child Health Research: Australia, 2009.

4. Department of Health. Research Governance Framework for Health and Social Care. 2nd Edition. London: Department of Health, 2005.

5. National Institute for Health Research (NIHR). Patients and Public. Last accessed 18/06/2012 at URL http://www.crncc.nihr.ac.uk/PI

6. Beresford P. User Involvement in research and evaluation: Liberation or Regulation? Social Policy & Society 2002;1(2):95-105.

7. Beresford P. User Involvement in research: Exploring the challenges. Nursing Times Research 2003;8(1):36-46.

8. Popay J, Attree P, Hornby D et al. Community engagement to address the wider social determinants of health: A review of evidence on impact, experience and process. London: NICE, 2007. Last accessed on 18/06/2012 at URL http://www.nice.org.uk/guidance/index.jsp?action=folder&o=34709

For peer review only

9. Wyatt K, Carter M, Mahtani V et al. The impact of consumer involvement in research: an evaluation of consumer involvement in the London Primary Care Studies Programme. Family Practice 2008;25(3):154-161.

10. Caron-Flinterman JF, Broerse JEW, Teerling J et al. Patients' priorities concerning health research: The case of asthma and COPD research in the Netherlands. Health Expect 2005;8(3):253-63.

11. Wright DNM, Corner JL, Hopkinson JB et al. The case for user involvement in research: the research priorities of cancer patients. Breast Cancer Research 2007;9(Suppl 2):S3.

12. Marsden J, Bradburn J. Patient and clinician collaboration in the design of a national randomised breast cancer trial. Health Expect 2004;7:6-17.

13. Hewlett S, de Wit M, Richards P et al. Patients and professionals as research partners: Challenges, practicalities, and benefits. Arthritis & Rheumatism 2006;55(4);676-680.

14. Minogue V, Boness J, Brown A et al. The impact of service user involvement in research. International Journal of Health Care Quality Assurance 2005;18(2):103-112.

15. Boote J, Telford R, Cooper C. Consumer involvement in health research: a review and research agenda. Health Policy 2002;61(2):213-236.

16. Staley K. Exploring impact: PI in NHS, public health and social care research. Eastleigh: INVOLVE, 2009.

For peer review only

17. Barber R, Boote JD, Parry GD et al.Can the impact of PI on research be evaluated? A mixed methods study. Health Expect 2011; 15(3):229-241.

18. Staniszewska S, Brett J, Mockford C et al. The GRIPP checklist: strengthening the quality of patient and PI reporting in research. International Journal of Technology Assessment in Health Care 2011;27(4):391–399.

19. Entwistle VA, Renfrew MJ, Yearley S et al. Lay perspectives: advantages for health researchers. BMJ 1998;316:463-466.

20. Oliver S, Milne R, Bradburn J et al. Investigating consumer perspectives on evaluating health technologies. Evaluation 2001;7:468.

21. Oliver SR, Rees RW, Clarke-Jones L et al. A multidimensional conceptual framework for analysing public involvement in health services research. Health Expect 2008;11:72-84.

22. Gradinger F, Britten N, Wyatt K et al. Values associated with Public Involvement in Health and Social Care Research. Health Expect, in press.

23. INVOLVE. INVOLVE Strategy 2012-2015. Putting people first in research. Eastleigh: INVOLVE 2012a. Last accessed 18/06/2012 at URL http://www.invo.org.uk

24. Duffield C. The Delphi Technique: a comparison of results obtained using two expert panels. International Journal of Nursing Studies 1993;30(3):227-237.

25. Hatton JM, Nunnelee JD. Research Priorities of Vascular Nursing. Journal of Vascular Nursing 1995;13(1):1-7.

26. Dalkey N, Helmer O. Delphi Technique: characteristics and sequence model to the use of experts. Management Science 1963;9(3):458-467.

For peer review only

27. Gallagher M, Hares T, Spencer J et al. The nominal group technique: a research tool for general practice. Family Practice 1993;10(1):76-81.

28. McKenna HP. The Delphi technique: a worthwhile approach for nursing? Journal of Advanced Nursing 1994;19(6);1221-1225.

29. Crisp J, Pelletier D, Duffield C et al. The Delphi Method? Nursing Research 1997;46(2):116-118.

30. Hassen F, Keeney S, McKenna H. Research Guidelines for the Delphi Survey Technique. Journal of Advanced Nursing 2000;32(4);1008-1015.

31. Jones J, Hunter D. Consensus methods for medical and health services research. BMJ 1995;311:276–380.

32. Walker A, Selfe J. The Delphi method: a useful tool for the allied health researcher. British Journal of Therapy and Rehabilitation 1996;3(12):677-681.

33. Keeney S, Hassona F, McKennab HP. A critical review of the Delphi technique as a research methodology for nursing. International Journal of Nursing Studies 2001;38(2):195-200.

34. Campbell SM, Braspenning J, Hutchinson A et al. Research methods used in developing and applying quality indicators in primary care. Qual Saf Health 2002;11:358–364.

35. Daykin N, Sanidas M, Barley V et al. Developing consensus and interprofessional working in cancer services: the case of user involvement. Journal of Interprofessional Care 2002;16(4): 405–406.

For peer review only

36. Shield T, Campbell S, Rogers A et al. Quality indicators for mental health care in primary care. Qual Saf Health 2003;12(2);100–106.

37. Boote J, Barber R, Cooper C. Principles and indicators of successful consumer involvement in NHS research: Results of a Delphi study and subgroup analysis. Health Policy 2006;75(3):280-297.

38. Efstathiou N, Ameen J, Coll AM. A Delphi study to identify healthcare users' priorities for cancer care in Greece. European Journal of Oncology Nursing 2008;12(3):262–371.

39. Lynn MR, Layman EL, Englebardt SP. Nursing administration research priorities: a National Delphi Study. Journal of Nursing Administration 1998;28(5):7-11.

40. Linstone HA, Turoff M. (eds). The Delphi Method: Techniques and Applications. Reading, Mass: Addison-Wesley, 1975.

41. Sackman H. Delphi Critique.). Lexington, Mass: Lexington Books, 1975.

42. McDonnell J, Meijler A, Kahan JP et al. Panellist consistency in the assessment of medical appropriateness. Health Policy 1996;37:139-152.

43. Hardy DJ, O'Brien AP, Gaskin CJ et al. Journal of Advanced Nursing 2004;46(1):95-109.

44. Lemmer B. Successive surveys of an expert panel: research in decision making with health visitors. Journal of Advanced Nursing 1998;27(3):538-545.

For peer review only

45. Green B, Jones M, Hughes D et al. Applying the Delphi technique in a study of GP's information requirement. Health and Social Care in the Community 1999;7(3):198-205.

46. Richie J, Spencer L. Qualitative data analysis for applied policy research. In: Bryman A, Burgess RG (Eds). Analysis Qualitative Data, London: Routledge, 1994, pp.173-194.

47. Boote J, Baird W, Beecroft C. Public involvement in the design stage of primary health research: A narrative review of case examples. Health Policy 2010; 95:10-23.

48. Hanley B, Bradburn J, Barnes M et al. Involving the public in NHS, public health and social care research: briefing notes for researchers, 2nd Edition. Eastleigh: INVOLVE, 2004.

49. INVOLVE. Briefing notes for researchers: involving the public in NHS, public health and social care research. Eastleigh: INVOLVE, 2012b Last accessed 8/07/2013 at URL http://www.invo.org.uk

50. Sitzia J, Wood N. Response rate in patient satisfaction research: an analysis of 210 published studies. Int J Qual Health Care, 1998;10:311–317.

51. Arber S. Designing samples. In: Gilbert N (ed). Researching Social Life. London: SAGE Publications, 2001.

52. Edwards P, Roberts I, Clarke M et al. Increasing response rates to postal questionnaires: systematic review. BMJ 2002;324:1183.

53. Beresford P, Campbell J. Disabled people, service users, user involvement and representation. Disability and Society 1994;9(3):315-25.

For peer review only

54. Morrow E, Boaz A, Brearley S et al. Handbook of service user involvement in nursing and health care research, Chpt 2, pp. 22-24. Chichester: Wiley Blackwell, 2012.

55. Abma TA, Widdershoven GAM. Sharing stories. Narrative and dialogue in responsive nursing evaluation. Evaluation and the Health Professions 2005;28:90-109.

56. McColl E, Jacoby A, Thomas L et al. Design and use of questionnaires: a review of best practice applicable to surveys of health service staff and patients. HTA 2001;5(31):101-132.

57. Peterson RA, Albaum G, Kerin RA. A note on alternative contact strategies in mail surveys. Journal of the Market Research Society 1989;31:409-418.

58. Boote J, Baird W, Sutton A. Public involvement in the systematic review process in health and social care: A narrative review of case examples. Health Policy 2011;102: 105-116.

For peer review only

Figure 1: What kinds of knowledge should inform health and social care research?*

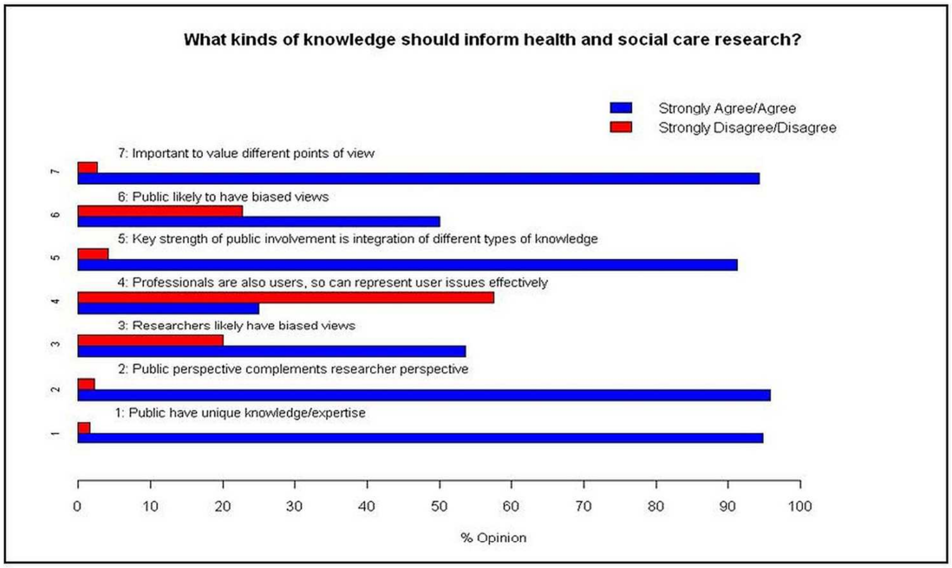

*Data taken from R1

120x90mm (300 x 300 DPI)

For peer review only - http://bmjopen.bmj.com/site/about/guidelines.xhtml

Figure 2: What are the purposes of public involvement in health and social care research?*

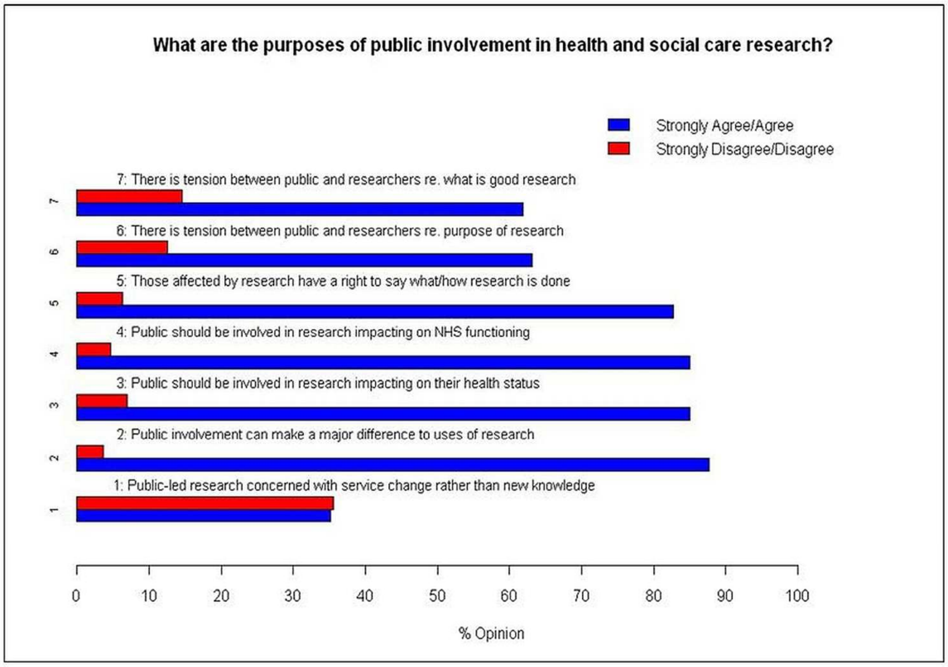

*Data taken from R1

106x90mm (300 x 300 DPI)

w only
